# Causality of Childhood and Adult Body Mass Index on Sick Sinus Syndrome: A Mendelian Randomization Study

**DOI:** 10.7759/cureus.80913

**Published:** 2025-03-20

**Authors:** Guanzhen Xu, Zhuang Liu, Ping Hou

**Affiliations:** 1 Graduate School, Affiliated Hospital of Liaoning University of Traditional Chinese Medicine, Shenyang, CHN; 2 Cardiology Department, Affiliated Hospital of Liaoning University of Traditional Chinese Medicine, Shenyang, CHN

**Keywords:** body mass index, causality, life course epidemiology, mendelian randomization, sick sinus syndrome

## Abstract

Background

The relationship between body mass index (BMI) and the risk of sick sinus syndrome (SSS) remains unclear. Clarifying the impact of BMI on SSS at different life stages is essential for advancing precision medicine and implementing effective prevention strategies to reduce the burden of SSS.

Methods

The causalities of childhood and adult BMI with SSS were investigated by univariate and multivariate Mendelian randomization. Reverse causalities were also explored to improve the accuracy of causality findings. Different sources of exposure data were used for replication analysis, and the effects of sample overlap were investigated using MRlap. The stability of the results was further enhanced through meta-analysis.

Results

There was a positive correlation of adult BMI with the risk of SSS in both the FinnGen (odds ratio (OR) = 1.17, 95% confidence interval (CI) 1.01-1.35, P = 0.031) and Integrative Epidemiology Unit (IEU) open genome-wide association study (GWAS) project (OR = 1.18, 95% CI 1.04-1.34, P = 0.009) databases. The causality remained valid after the correction of telomere length. There was no causality detected between childhood BMI and SSS, as determined by independent studies of the Early Growth Genetics (EGG) 2020 (OR = 1.06, 95% CI 0.89-1.27, P = 0.513) and EGG2015 (OR = 1.02, 95% CI 0.97-1.09, P = 0.423). Meta-analysis results further confirmed the reliability of the causal inference.

Conclusions

The findings indicate that elevated BMI in adults, particularly among middle-aged and elderly populations, increases the risk of developing SSS. In contrast, no causal relationship was observed between childhood BMI and SSS, suggesting that the influence of BMI on SSS susceptibility may predominantly emerge during later life stages. These results highlight the need for targeted public health interventions to address adult obesity as a modifiable risk factor for SSS.

## Introduction

Sick sinus syndrome (SSS) collectively refers to diseases caused due to abnormal cardiac impulses and conduction that are clinically manifested as sinus bradycardia, sinus arrest, and other arrhythmias [1]. Owing to the complex etiology and pathogenesis of SSS, there are currently no effective long-term therapeutic strategies targeting the underlying pathways. With advancing age, the increase in comorbidities is associated with a poorer prognosis observed in studies of patients with SSS. Permanent pacemaker implantation is considered the only effective treatment among patients with irreversible etiology [2]. In the United States, new SSS cases are expected to rise from 172,000 patients per year by 2060, which will undoubtedly impose a tremendous burden on the healthcare system [3,4]. Therefore, it is worth exploring the risk factors of SSS to facilitate early disease management and prevent progression.

Studies have highlighted high body mass index (BMI), age, and low heart rate as risk factors for SSS [4]. In particular, age is considered as a major risk factor, as aging is accompanied by a gradual decline in the sinoatrial conduction time and high fibrosis [5,6]. Although the onset of SSS is predominately reported among the elderly population, SSS is regarded as a disease at all ages [7].

BMI is an indirect measure of body fat. Research findings have projected that more than half of the global population will be overweight (BMI ≥ 30 kg/m^2^) or obese (BMI ≥ 25 kg/m^2^) by 2030 [8]. Elevated BMI increases the risk of atrial fibrillation, ischemic stroke, and type 2 diabetes mellitus [9-11] and was found to significantly raise the worldwide disease burden [12]. To date, no randomized controlled trial has been performed to investigate the causality between BMI and SSS. Clarification of the relationship between BMI and SSS is essential for promoting early disease prevention strategies and precision treatment.

When observational studies are scarce, Mendelian randomization (MR) serves as an intuitive method for inferring causality. Considering the fact that genetic variation is already determined at the time of conception, MR equivalently assesses the relationship between exposure and the level of genetic prediction of the outcome. In addition, it avoids any interference from reverse causality and confounders in observational studies and provides researchers with valid etiological insight [13,14].

## Materials and methods

Study overview

**Figure 1:**
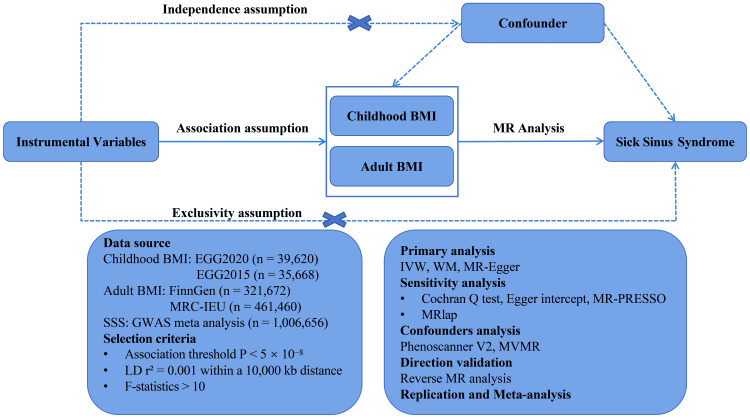
Overview of the Mendelian randomization study The extraction of instrumental variables (IVs) adheres to three core assumptions: (1) the IVs are strongly associated with the exposure (relevance assumption), (2) the IVs are independent of confounders (independence assumption), and (3) the IVs have no direct effect on the outcome, influencing the outcome only through the exposure (exclusion restriction assumption). BMI, body mass index; EGG, Early Growth Genetics; FinnGen, Finnish Health Research Environment for Genomic Research; GWAS, Genome-Wide Association Study; IVW, inverse-variance weighted; LD, linkage disequilibrium; MR, Mendelian randomization; MRC-IEU, Medical Research Council Integrated Epidemiology Unit; MR-PRESSO, Mendelian Randomization - Pleiotropy Residual Sum and Outlier; MVMR, multivariate Mendelian randomization; SSS, sick sinus syndrome; WM, weighted median

This study is reported in accordance with the Strengthening the Reporting of Observational Studies in Epidemiology using Mendelian Randomization (STROBE-MR) statement [15]. This study explored the causalities of childhood and adult BMI on SSS. To enhance the robustness of the causal inference, replication analyses were conducted using different exposure data sources. The Phenoscanner database and multivariate Mendelian randomization (MVMR) were used to examine any interference from confounding factors. In addition, MR results were meta-merged. The study overview is shown in Figure 1. All analyses were performed using the “TwoSampleMR” package (0.6.5), “MendelianRandomization” package (0.10.0), “MVMR” package (0.4), and “meta” (8.0-1) in the R program (4.3.2; R Development Core Team, Vienna, Austria).

Data source

Childhood BMI data were derived from a meta-analysis of genome-wide association studies (GWAS) of BMI conducted among 39,620 children between two and 10 years of age from 26 studies and updated by the Early Growth Genetics (EGG) consortium [16]. Another meta-analysis of genome-wide association studies of BMI in 35,668 children of 2-10 years from earlier studies was included in the replication analysis [17].

The data on adult BMI were obtained from the FinnGen study where approximately 500,000 participants with a median age of 53 years old were enrolled [18]. The Integrative Epidemiology Unit (IEU) open GWAS database (IEU Open GWAS project (mrcieu.ac.uk)) carrying genetic and health information from approximately 500,000 European individuals aged 40-69 years was employed for replication analysis [19]. While sample overlapping may have occurred between exposure and outcome in the replication analysis, efforts were taken to use non-overlapping samples in line with the STROBE statement recommendation. The direct calculation of the rate of sample overlap is challenging. We thus employed the MRlap method to correct for potential winner’s curse and weak instrument bias. A detailed description of the MRlap method is provided in the subsequent section.

Telomere length (TL) has been incorporated into aging-related proxies, and telomere shortening has been observed during normal aging in humans and mice [20]. TL data were derived from the IEU OPEN GWAS database harboring genetic information of 472,174 European individuals.

SSS summary data were obtained from the largest meta-analysis available where SSS diagnoses were conducted in accordance with the International Classification of Diseases, 9th (ICD-9: 427.8) or 10th (ICD-10: I49.5) editions [6]. Table 1 shows specific information on data sources.

**Table 1:** Sources of GWAS data used in the MR study BMI, body mass index; CHB-CVS, Copenhagen Hospital Biobank Cardiovascular Study; DBDS, Danish Blood Donor Study; EGG, Early Growth Genetics; FinnGen, Finnish Health Research Environment for Genomic Research; GWAS, genome-wide association studies; HUNT, Norwegian Nord-Trøndelag Health Study; MR, Mendelian randomization; MRC-IEU, Medical Research Council Integrated Epidemiology Unit; NA, not applicable; SSS, sick sinus syndrome; TL, telomere length; UKB, UK Biobank

Phenotypes	Consortium	Ancestry	PMID	Sample size	Web source
Adult BMI	FinnGen	European	NA	321,672	https://r11.finngen.fi/
Adult BMI	MRC-IEU	European	NA	461,460	https://gwas.mrcieu.ac.uk/datasets/ukb-b-19953/
Childhood BMI	EGG	European	33045005	39,620	http://egg-consortium.org/
Childhood BMI	EGG	European	26604143	35,668	http://egg-consortium.org/
SSS	The deCODE Genetics,UKB, the CHB-CVS/DBDS, the HUNT cohort	European	33580673	1,006,656 (6469 cases and 1,000,187 controls)	https://www.decode.com
TL	NA	European	NA	472,174	https://gwas.mrcieu.ac.uk/datasets/ieu-b-4879/

Selection of instrumental variables (IVs)

The IVs were extracted in line with the assumptions of association, independence, and exclusivity in MR [21]. Single nucleotide polymorphisms (SNPs) were critically screened as follows. (a) To identify exposure-associated and independent SNPs, a threshold of P < 5 × 10^−8^ was set with the removal of SNPs that were in linkage disequilibrium (LD) (r² = 0.001, kb = 10,000). (b) Exposure-associated SNPs were extracted from the outcome dataset, and SNPs associated with the outcome phenotype were removed (P < 5 × 10^−8^). (c) Alleles were harmonized for exposure and outcome, and palindromic and incompatible SNPs were removed. (d) Building on previous findings that identified age-related ion channel disorders and idiopathic fibrosis as major risk factors for SSS [22-25], we employed univariate MR (UVMR) analysis to evaluate aging-related phenotypic SNPs through the PhenoScanner database [26]. (e) SNPs with F < 10 were eliminated, as they are in general considered to represent weak instrumental variables [27]. Additionally, statistical power for the estimates derived from the inverse variance weighted (IVW) method was calculated using an online computational tool (https://sb452.shinyapps.io/power/). After the above screening steps, any potential horizontal pleiotropy outliers were determined by MR-Pleiotropy Residual Sum and Outlier (MR-PRESSO) [28]. The screened SNPs were finally used in the MR study.

UVMR analysis

In UVMR, the causality of childhood and adult BMI with SSS and the reverse causality of SSS with BMI were separately examined. The IVW method was employed for the main analysis and provided robust causal inference under the hypothesis that all IVs were valid [14]. A value of P < 0.05 in the IVW method indicated significant causality between exposure and outcome data. In addition, the weighted median method and the MR-Egger method served as supplements, wherein the former allowed for a more precise estimation if half of the IVs were valid [29]. Although the MR-Egger method had lower statistical performance, it alleviated the reliability of the causal inference when the direction of the MR-Egger estimate was consistent with that of the other methods.

During sensitivity analysis, a Cochran’s Q test was employed for heterogeneity analysis. A value of P < 0.05 indicated the presence of heterogeneity. A random-effect IVW method analysis was performed to mitigate any effect of heterogeneity. In addition, horizontal pleiotropy was assessed using the MR-Egger intercept. An intercept value of P < 0.05 indicated potential pleiotropy in the results and weakened the validity of the causal inference conclusion.

MVMR analysis

MVMR, as an extension of UVMR, is more advantageous than UVMR when several relevant exposures have common genetic predictors. Based on previous studies, we included adult BMI, TL, and SSS in the MVMR model to differentiate the effects of confounding factors (TL) and to examine the independent causal relationship between adult BMI and SSS [25]. The shortening and damage of TL are recognized causes of aging and cellular senescence [30-32]. Epidemiological data on TL have demonstrated that shorter TL is associated with age [33]. In healthy newborns, median leukocyte TLs (LTLs) ranged from 8.5 to 13.5 kb. LTL was observed to shorten at an average annual rate of 30-35 bp, reaching about 5-6 kb in people over 60 years old [30]. Similarly, three complementary analysis methods, IVW, weighted median, and MR-Egger, were performed. Cochran's Q test was conducted for heterogeneity analysis, and random-effect IVW results were used for causal inference once heterogeneity was detected in MVMR.

MRlap

The MRlap method addresses the issues of weak IVs bias and winner’s curse arising from sample overlap and is beneficial in situations where the use of overlapping samples is unavoidable. In particular, the method adjusts for causality estimates using linkage disequilibrium score regression and other techniques based on the hypothesis that the exposure adopts a spike-and-slab genomic structure. The effect estimates can be used as sensitivity analysis. Herein, we employed strict thresholds (MR threshold  =  5 × 10 −8 , MR pruning distribution = 10,000, MR pruning LD  =  0.001). The bias did not affect the results for non-overlapping samples. In the case of sample overlapping, corrected results were used if they were statistically significant compared to the IVW results (P < 0.05) because the bias of the corrected results was small and unrelated to sample overlapping. In the absence of any significant difference(P > 0.05), the IVW-MR estimates were used [34,35].

Meta-analysis

In meta-analysis, MR estimates were combined using a fixed-effects meta-analysis. Heterogeneity across different data sources was assessed by the I² statistic, with I² values of <25%, 25-75%, and >75%, indicating low, moderate, and high heterogeneity, respectively. The meta-analysis of IVW methods in UVMR and MVMR results further increased the robustness and generalizability of causal inference.

## Results

UVMR and meta-analysis

**Figure 2:**
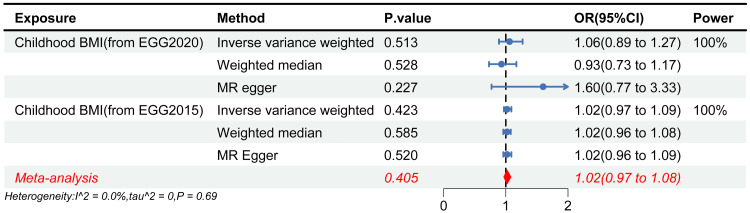
Forest plot of the MR causal estimates for childhood BMI and SSS BMI, body mass index; CI, confidence interval; EGG, Early Growth Genetics; MR, Mendelian randomization; OR, odds ratio; SSS, sick sinus syndrome

In the MR analysis, the causal relationship between childhood BMI and SSS was not supported (Figure 2). This was observed in both the analyses based on the EGG (2020) (odds ratio (OR) = 1.06, 95% confidence interval (CI) 0.89-1.27, P = 0.513) and the replication analyses using the EGG (2015) (OR = 1.02, 95% CI 0.97-1.09, P = 0.423). In reverse MR, no association was detected between SSS susceptibility and childhood BMI. No outliers, heterogeneity, or horizontal pleiotropy were recorded in all the above analyses. The meta-analysis found no evidence supporting a causal relationship between childhood BMI and the risk of SSS (OR = 1.02, 95% CI 0.97-1.08, P = 0.405).

**Figure 3:**
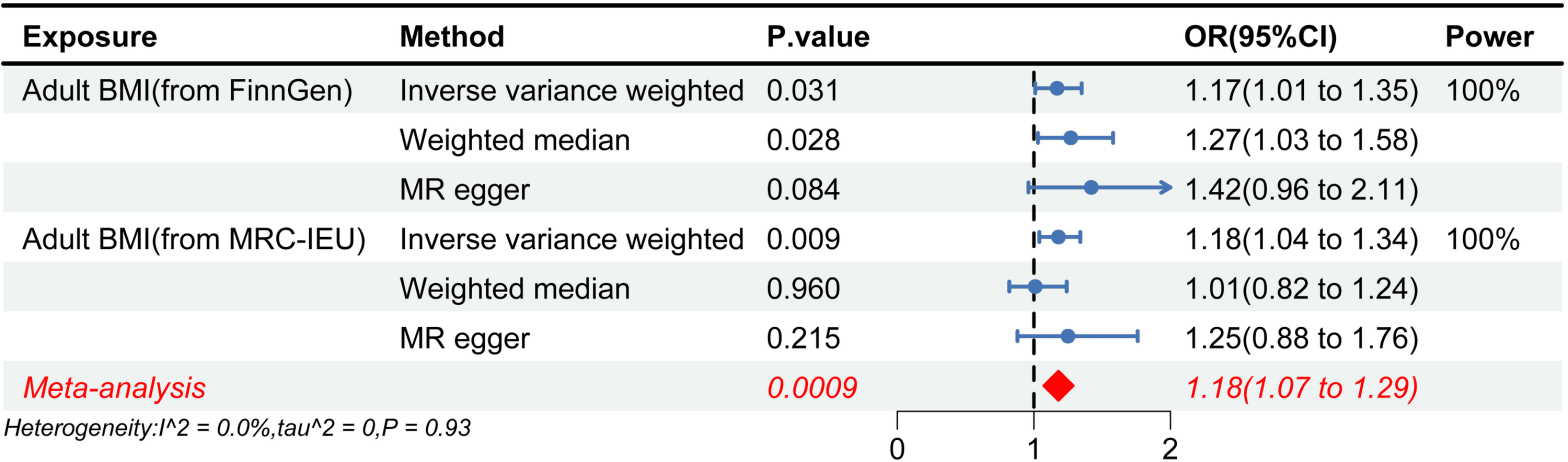
Forest plot of the MR causal estimates for adult BMI and SSS BMI, body mass index; CI, confidence interval; FinnGen, Finnish Health Research Environment for Genomic Research; MR, Mendelian randomization; OR, odds ratio; MRC-IEU, Medical Research Council Integrated Epidemiology Unit; SSS, sick sinus syndrome

Adult BMI showed a positive association with SSS, which demonstrated a consistent causal inference across different sources of the BMI database (Figure 3). During the analysis of FinnGen’s adult BMI data, outliers were screened and eliminated by MR-PRESSO (rs914187). The results showed that adult BMI elevated the risk of SSS (OR = 1.17, 95% CI 1.01-1.35, P = 0.031) without any heterogeneity (P_Cochran’s Q_ = 0.13) and horizontal pleiotropy (P_Intercept_ = 0.30). As per reverse MR results, the identified causality was not affected by reverse causality (OR = 1.01, 95% CI 0.99-1.03, P = 0.397). In the replication analysis, we excluded the SNP (rs2870111) associated with aging. The causality conclusion was consistent in the adult BMI data of IEU OPEN GWAS (OR = 1.18, 95% CI 1.04-1.34, P = 0.009) and showed no heterogeneity (P_Cochran’s Q_ = 0.07) and horizontal pleiotropy (P_Intercept_ = 0.74). Similarly, causality was not affected by reverse causality (OR = 0.99, 95% CI 0.98-1.01, P = 0.373). MRlap analyses supported the causality between adult BMI and SSS. Sample overlapping did not affect MR results, while the difference between observed and corrected effects was statistically insignificant and in agreement with the findings of the IVW results. This observation further confirms that the causal inference was robust (Observed_effect_p = 0.014, Corrected_effect_p = 0.029, P_difference = 0.667). The meta-analysis further corroborated the causal relationship between elevated adult BMI and an increased risk of sick sinus syndrome SSS (OR = 1.18, 95% CI 1.07-1.29, P = 0.0009).

**Figure 4:**
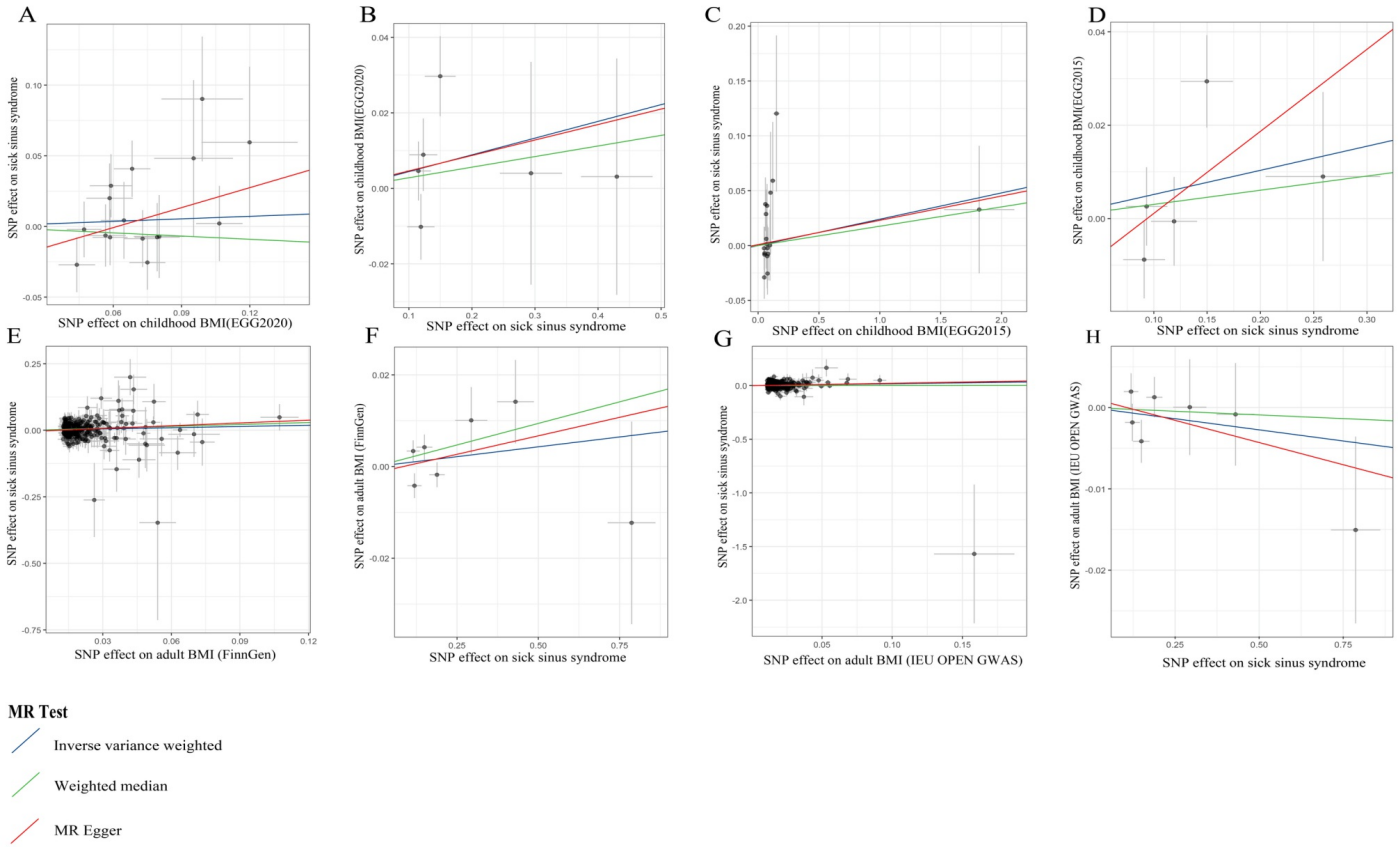
Scatter plot depicting causal associations between childhood/adult BMI and SSS (A) Between childhood BMI (EGG2020) and SSS. (B) Between SSS and childhood BMI (EGG2020). (C) Between childhood BMI (EGG2015) and SSS. (D) Between SSS and childhood BMI (EGG2015). (E) Between adult BMI (FinnGen) and SSS. (F) Between SSS and adult BMI (FinnGen). (G) Between adult BMI (IEU OPEN GWAS) and SSS. (H) Between SSS and adult BMI (IEU OPEN GWAS). BMI, body mass index; EGG, Early Growth Genetics; FinnGen, Finnish Health Research Environment for Genomic Research; IEU OPEN GWAS, Integrative Epidemiology Unit Open Genome-Wide Association Study; MR, Mendelian randomization; SNP, single nucleotide polymorphism; SSS, sick sinus syndrome

The visualized scatterplot is shown in Figure 4, and the detailed results of IVs and MR analysis are described in Tables 2-11.

**Table 2:** The Reverse MR analysis does not support a causal relationship between SSS and BMI BMI, body mass index; CI, confidence interval; EGG, Early Growth Genetics; FinnGen, Finnish Health Research Environment for Genomic Research; IEU OPEN GWAS, Integrative Epidemiology Unit Open Genome-Wide Association Study; IVW, inverse-variance weighted; MR, Mendelian randomization; OR, odds ratio; SSS, sick sinus syndrome; WM, weighted median

Exposure	Outcome	IVW		WM		MR-Egger		Power
		OR (95 % CI)	p	OR (95 % CI)	p	OR (95 % CI)	p	
SSS	Childhood BMI (EGG2020)	1.05 (0.97, 1.13)	0.266	1.03 (0.95, 1.11)	0.492	1.04 (0.81, 1.33)	0.762	3.3%
	Childhood BMI (EGG2015)	1.05 (0.96, 1.16)	0.294	1.03 (0.94, 1.14)	0.537	1.19 (0.88, 1.62)	0.340	3.2%
	Adult BMI (FinnGen)	1.01 (0.99, 1.03)	0.397	1.02 (1.00, 1.04)	0.080	1.02 (0.96, 1.07)	0.586	3.2%
	Adult BMI (IEU OPEN GWAS)	0.99 (0.98, 1.01)	0.373	1.00 (0.98, 1.01)	0.827	0.99 (0.96, 1.01)	0.434	3.4%

**Table 3:** Sensitivity analysis of the Mendelian randomization BMI, body mass index; EGG, Early Growth Genetics; FinnGen, Finnish Health Research Environment for Genomic Research; IEU OPEN GWAS, Integrative Epidemiology Unit Open Genome-Wide Association Study; MR-PRESSO, Mendelian randomization pleiotropy residual sum and outlier; NA, not applicable; SSS, sick sinus syndrome

Exposure	Outcome	Heterogeneity		Pleiotropy		MR-PRESSO	
		Cochran Q	P	Intercept	P	Outlier-corrected	P
Childhood BMI (EGG2020)	SSS	16.58	0.34	-0.03	0.27	NA	0.33
Childhood BMI (EGG2015)		16.45	0.49	0.001	0.90	NA	0.627
Adult BMI (FinnGen)		311.48	0.13	-0.004	0.30	rs914187	0.048
						NA	0.12
Adult BMI (IEU OPEN GWAS)		416.15	0.07	-0.001	0.74	NA	0.06
SSS	Childhood BMI (EGG2020)	8.38	0.14	0.001	0.98	NA	0.16
	Childhood BMI (EGG2015)	8.07	0.09	-0.02	0.46	NA	0.17
	Adult BMI (FinnGen)	10.61	0.10	-0.001	0.78	NA	0.14
	Adult BMI (IEU OPEN GWAS)	5.07	0.54	0.001	0.65	NA	0.52

**Table 4:** Independent IVs in the causal analysis of childhood BMI (EGG2020) and SSS BMI, body mass index; EGG, early growth genetics; IVs, instrumental variables; NA, not applicable; SNP: single nucleotide polymorphism; SSS, sick sinus syndrome

SNP	effect_allele.exposure	other_allele.exposure	effect_allele.outcome	other_allele.outcome	beta.exposure	beta.outcome	eaf.exposure	eaf.outcome	pval.outcome	se.outcome	samplesize.outcome	chr.exposure	pos.exposure	se.exposure	pval.exposure	samplesize.exposure	R2	F
rs114670539	T	C	T	C	0.0991	0.09019713	0.0487	NA	0.0411	0.0441	1006656	2	207064335	0.0179	3.16E-08	39618	0.000773059	30.64921265
rs11676272	A	G	A	G	-0.075	0.02550244	0.5358	NA	0.184	0.0192	1006656	2	25141538	0.0079	2.37E-21	36714	0.002448904	90.12487706
rs12042908	A	G	A	G	0.0586	-0.007699566	0.4523	NA	0.694	0.0197	1006656	1	74997762	0.0077	2.77E-14	38693	0.001494623	57.9150363
rs12641981	T	C	T	C	0.044	-0.027104018	0.4185	NA	0.162	0.0194	1006656	4	45179883	0.008	4.19E-08	34819	0.000868024	30.24826244
rs13107325	T	C	T	C	0.0953	0.048199514	0.0795	NA	0.383	0.0553	1006656	4	103188709	0.0173	3.51E-08	36677	0.000826686	30.34379616
rs17817449	T	G	T	G	-0.0683	-0.040796394	0.5855	NA	0.0399	0.0199	1006656	16	53813367	0.008	1.69E-17	36963	0.001968061	72.88496237
rs41279738	T	G	T	G	-0.1199	-0.059503994	0.9692	NA	0.266	0.0535	1006656	1	110082551	0.0211	1.30E-08	39618	0.00081438	32.28877219
rs4477562	T	C	T	C	0.0802	-0.007095111	0.1103	NA	0.81	0.0293	1006656	13	54104968	0.0112	8.29E-13	39618	0.001292583	51.27324057
rs543874	A	G	A	G	-0.0793	0.007598798	0.8131	NA	0.752	0.0241	1006656	1	177889480	0.0099	1.62E-15	36963	0.001732828	64.15824652
rs56133711	A	G	A	G	0.0566	-0.006400439	0.2296	NA	0.77	0.022	1006656	11	27723334	0.0089	2.00E-10	38160	0.001058728	40.44176365
rs571312	A	C	A	C	0.059	0.028801234	0.2396	NA	0.198	0.0223	1006656	18	57839769	0.0093	2.00E-10	37068	0.001084595	40.2452559
rs61765651	T	C	T	C	-0.0584	-0.019998647	0.174	NA	0.416	0.0245	1006656	1	72754314	0.0102	9.50E-09	38693	0.000846496	32.77954356
rs62500888	A	G	A	G	0.0472	-0.002097798	0.5408	NA	0.916	0.0197	1006656	8	28061823	0.0076	6.91E-10	39618	0.000972617	38.56868999
rs7138803	A	G	A	G	0.0729	-0.008596847	0.338	NA	0.669	0.0201	1006656	12	50247468	0.008	7.12E-20	37067	0.002235197	83.03317584
rs7199285	T	C	T	C	-0.0647	-0.004299228	0.171	NA	0.874	0.0272	1006656	16	19980931	0.0101	1.34E-10	37743	0.001086069	41.03390039
rs939584	T	C	T	C	0.1066	0.002102208	0.83	NA	0.937	0.0266	1006656	2	621558	0.0102	8.85E-26	38668	0.002816679	109.2173419
Summary	NA	NA	NA	NA	NA	NA	NA	NA	NA	NA	NA	NA	NA	NA	NA	NA	0.022319532	845.2060775

**Table 5:** Independent IVs in the causal analysis of childhood BMI (EGG2015) and SSS BMI, body mass index; EGG, Early Growth Genetics; IVs, instrumental variables; NA, not applicable; SNP: single nucleotide polymorphism; SSS, sick sinus syndrome

SNP	effect_allele.exposure	other_allele.exposure	effect_allele.outcome	other_allele.outcome	beta.exposure	beta.outcome	eaf.exposure	eaf.outcome	pval.outcome	se.outcome	samplesize.outcome	chr.exposure	pos.exposure	se.exposure	pval.exposure	samplesize.exposure	R2	F
rs11676272	A	G	A	G	-0.0769	0.02550244	0.0288	NA	0.184	0.0192	1006656	2	24995042	0.0078	8.55E-23	35455.7	0.002733937	97.19389255
rs12041852	A	G	A	G	-0.0525	0.008203557	NA	NA	0.678	0.0197	1006656	1	74776088	0.0082	1.77E-10	31111	0.001315846	40.9885903
rs12429545	A	G	A	G	0.0745	-0.009696863	NA	NA	0.738	0.0291	1006656	13	53000207	0.0113	3.66E-11	35646	0.001217911	43.46416
rs13107325	T	C	T	C	0.1022	0.048199514	NA	NA	0.383	0.0553	1006656	4	103407732	0.0179	1.19E-08	35238.5	0.000924223	32.5965082
rs13130484	T	C	T	C	0.0506	-0.02899636	NA	NA	0.138	0.0195	1006656	4	44870448	0.0078	8.94E-11	35656.7	0.00117885	42.08113722
rs13253111	A	G	A	G	0.0492	-0.00249688	NA	NA	0.9	0.0197	1006656	8	28117893	0.0084	4.13E-09	30118	0.001137761	34.30384433
rs13387838	A	G	A	G	0.1507	0.120304299	NA	NA	0.091	0.0712	1006656	2	206989692	0.027	2.40E-08	26511.8	0.00117368	31.15058541
rs1421085	T	C	T	C	-0.0734	-0.036302999	NA	NA	0.0673	0.0198	1006656	16	52358455	0.0082	3.20E-19	33427	0.002391263	80.11953677
rs17309930	A	C	A	C	0.0529	-0.007004474	NA	NA	0.769	0.0239	1006656	11	27705069	0.0095	2.47E-08	35532.6	0.000871883	31.00556773
rs2590942	T	G	T	G	0.0598	0.037701867	NA	NA	0.123	0.0244	1006656	1	72657869	0.0102	3.88E-09	35652	0.000963163	34.3698519
rs4854349	T	C	T	C	-0.0974	-0.000300045	NA	NA	0.992	0.0323	1006656	2	637861	0.0104	6.00E-21	34909.9	0.002506183	87.70540403
rs4870949	T	C	T	C	1.8167	0.032796274	NA	NA	0.572	0.0581	1006656	8	126704776	0.2887	3.13E-10	923	0.041136556	39.512162
rs543874	A	G	A	G	-0.0819	0.007598798	NA	NA	0.752	0.0241	1006656	1	176156103	0.0097	2.38E-17	35668	0.001994704	71.2853001
rs6567160	T	C	T	C	-0.0649	-0.028704068	NA	NA	0.198	0.0223	1006656	18	55980115	0.0094	4.06E-12	35664.9	0.001334789	47.66606837
rs7132908	A	G	A	G	0.0768	-0.002302649	NA	NA	0.908	0.02	1006656	12	48549415	0.0086	4.99E-19	29915.4	0.002658732	79.7437219
rs7550711	T	C	T	C	0.12	0.059098752	NA	NA	0.269	0.0535	1006656	1	109884409	0.0212	1.50E-08	33434	0.000957385	32.03795524
rs8046312	A	C	A	C	0.0668	0.006098558	NA	NA	0.82	0.027	1006656	16	19886835	0.0107	4.06E-10	30085.9	0.001293779	38.9723414
rs987237	A	G	A	G	-0.0712	-0.001399021	NA	NA	0.956	0.0252	1006656	6	50911009	0.0098	3.81E-13	35663	0.001477909	52.78171286
Summary	NA	NA	NA	NA	NA	NA	NA	NA	NA	NA	NA	NA	NA	NA	NA	NA	0.067268552	916.9783403

**Table 6:** Independent IVs in the causal analysis of SSS and childhood BMI (EGG2020) BMI, body mass index; EGG, Early Growth Genetics; IVs, instrumental variables; NA, not applicable; SNP, single nucleotide polymorphism; SSS, sick sinus syndrome

SNP	effect_allele.exposure	other_allele.exposure	effect_allele.outcome	other_allele.outcome	beta.exposure	beta.outcome	eaf.exposure	eaf.outcome	chr.outcome	pos.outcome	se.outcome	pval.outcome	samplesize.outcome	pval.exposure	se.exposure	samplesize.exposure	R2	F
rs10149621	G	T	G	T	0.429799933	0.0031	NA	NA	14	23980080	0.0313	0.9215	35922	3.45E-14	0.0567	1006656	5.71E-05	57.46001123
rs116307579	T	C	T	C	0.293698935	0.004	NA	NA	14	24534471	0.0295	0.8919	36235	2.78E-09	0.0494	1006656	3.51E-05	35.34679019
rs11662320	T	C	T	C	0.119000073	-0.0102	NA	NA	18	67177899	0.0087	0.2386	39722	3.68E-08	0.0216	1006656	3.02E-05	30.35191429
rs11711941	T	C	T	C	0.115103894	0.0046	NA	NA	3	38778540	0.0078	0.5558	38160	6.93E-09	0.0199	1006656	3.32E-05	33.4559233
rs2106261	T	C	T	C	0.149703664	0.0297	NA	NA	16	73051620	0.0106	0.005114	35505	9.94E-10	0.0245	1006656	3.71E-05	37.33634715
rs35813871	A	G	A	G	0.122898817	0.0089	NA	NA	2	179650408	0.0096	0.353	37034.9	2.64E-08	0.0221	1006656	3.07E-05	30.92502019
Summary	NA	NA	NA	NA	NA	NA	NA	NA	NA	NA	NA	NA	NA	NA	NA	NA	0.000223381	224.8760064

**Table 7:** Independent IVs in the causal analysis of SSS and childhood BMI (EGG2015) BMI, body mass index;EGG, Early Growth Genetics; IVs, instrumental variables; NA, not applicable; SNP, single nucleotide polymorphism; SSS, sick sinus syndrome

SNP	effect_allele.exposure	other_allele.exposure	effect_allele.outcome	other_allele.outcome	beta.exposure	beta.outcome	eaf.exposure	eaf.outcome	chr.outcome	pos.outcome	se.outcome	pval.outcome	samplesize.outcome	pval.exposure	se.exposure	samplesize.exposure	R2	F
rs1009360	C	T	C	T	-0.093201405	-0.0026	NA	NA	2	65129553	0.0084	0.754	30111	1.69E-06	0.0195	1006656	2.27E-05	22.84414097
rs11662320	T	C	T	C	0.119000073	-6.00E-04	NA	NA	18	65328879	0.0095	0.9526	30106.8	3.68E-08	0.0216	1006656	3.02E-05	30.35191429
rs13023947	T	C	T	C	-0.258705964	-0.009	NA	NA	2	129680524	0.0181	0.617	29990	1.51E-06	0.0538	1006656	2.30E-05	23.1231751
rs1867785	A	G	A	G	0.090900471	-0.0088	NA	NA	2	46387842	0.0083	0.2903	29915	3.75E-06	0.0196	1006656	2.14E-05	21.5089527
rs2106261	T	C	T	C	0.149703664	0.0294	NA	NA	16	71609121	0.0099	0.003091	35634	9.94E-10	0.0245	1006656	3.71E-05	37.33634715
Summary	NA	NA	NA	NA	NA	NA	NA	NA	NA	NA	NA	NA	NA	NA	NA	NA	0.000134267	135.1645302

**Table 8:** Independent IVs in the causal analysis of adult BMI (FinnGen) and SSS BMI, body mass index; FinnGen, Finnish Health Research Environment for Genomic Research; IVs, instrumental variables; NA, not applicable; SNP, single nucleotide polymorphism; SSS, sick sinus syndrome

SNP	effect_allele.exposure	other_allele.exposure	effect_allele.outcome	other_allele.outcome	beta.exposure	beta.outcome	eaf.exposure	eaf.outcome	pval.outcome	se.outcome	samplesize.outcome	pval.exposure	se.exposure	samplesize.exposure	R2	F
rs1000940	G	A	G	A	0.0155263	0.026797712	0.42681	NA	0.194	0.0207	1006656	4.01E-11	0.0023511	321672	0.000135557	43.61049775
rs10269783	A	G	A	G	0.0147676	-0.007004474	0.458426	NA	0.724	0.0198	1006656	2.02E-10	0.00232208	321672	0.000125718	40.44480862
rs10275348	C	T	C	T	-0.0137198	0.010900374	0.332516	NA	0.59	0.0202	1006656	2.51E-08	0.00246194	321672	9.65E-05	31.05545751
rs10404037	A	G	A	G	-0.0194287	-0.00950503	0.131998	NA	0.722	0.0266	1006656	1.34E-08	0.00341988	321672	0.000100325	32.2747621
rs10456701	T	C	T	C	0.0225431	0.021702782	0.283021	NA	0.289	0.0205	1006656	2.40E-18	0.00258023	321672	0.000237243	76.332192
rs10757687	C	T	C	T	0.0176	-0.034105021	0.498315	NA	0.0803	0.0195	1006656	2.86E-14	0.00231447	321672	0.000179734	57.82551691
rs10769936	C	T	C	T	0.0162783	-0.015095488	0.74628	NA	0.5	0.0224	1006656	9.64E-10	0.00266189	321672	0.000116245	37.39686412
rs10787738	T	C	T	C	0.0166963	0.001598721	0.256679	NA	0.943	0.0227	1006656	3.33E-10	0.00265746	321672	0.000122699	39.47337917
rs10846920	T	C	T	C	0.0189662	-0.013902905	0.76721	NA	0.535	0.0224	1006656	5.47E-12	0.00275163	321672	0.000147674	47.50921779
rs10859055	T	C	T	C	-0.0149815	-0.011000282	0.364881	NA	0.582	0.02	1006656	4.77E-10	0.00240614	321672	0.000120504	38.76734964
rs10929925	A	C	A	C	-0.0195152	0.028295869	0.496719	NA	0.15	0.0197	1006656	3.69E-17	0.0023171	321672	0.000220469	70.93388692
rs10947793	G	A	G	A	-0.0131812	-0.012295278	0.388344	NA	0.543	0.0202	1006656	3.14E-08	0.00238222	321672	9.52E-05	30.61564872
rs10987749	C	A	C	A	-0.0145852	0.023104874	0.569461	NA	0.25	0.0201	1006656	4.38E-10	0.00233743	321672	0.000121027	38.93541874
rs11038839	G	A	G	A	0.0433566	0.07289724	0.0406188	NA	0.212	0.0584	1006656	2.31E-13	0.00591515	321672	0.000166991	53.72497361
rs1106236	C	T	C	T	-0.0133559	-0.046096296	0.401313	NA	0.0241	0.0204	1006656	1.84E-08	0.00237379	321672	9.84E-05	31.65621779
rs11098675	G	A	G	A	-0.0173683	-0.017997077	0.826396	NA	0.463	0.0246	1006656	1.31E-08	0.00305543	321672	0.000100441	32.31225346
rs11134425	A	G	A	G	-0.014844	0.011202515	0.412076	NA	0.563	0.0194	1006656	2.90E-10	0.00235476	321672	0.000123521	39.73798956
rs11150745	G	A	G	A	-0.0197904	0.001898197	0.281538	NA	0.927	0.0209	1006656	1.59E-14	0.00257682	321672	0.000183336	58.98455114
rs11159989	T	G	T	G	-0.0151418	0.053597637	0.289069	NA	0.00996	0.0208	1006656	3.07E-09	0.00255446	321672	0.000109218	35.13614396
rs11165493	A	G	A	G	0.0155747	-0.020600746	0.378985	NA	0.312	0.0204	1006656	7.29E-11	0.00239076	321672	0.000131916	42.43895855
rs11170433	T	C	T	C	-0.015324	0.027104018	0.562273	NA	0.177	0.0201	1006656	6.33E-11	0.00234462	321672	0.000132779	42.71659782
rs11185111	A	G	A	G	-0.0175914	-0.014798967	0.42458	NA	0.486	0.0212	1006656	7.47E-14	0.00235202	321672	0.000173872	55.93917883
rs11186668	G	A	G	A	-0.013075	-0.023596214	0.431816	NA	0.264	0.0212	1006656	2.35E-08	0.00234137	321672	9.69E-05	31.18463003
rs11192614	C	T	C	T	-0.0150153	0.013695783	0.353292	NA	0.506	0.0205	1006656	6.20E-10	0.0024276	321672	0.000118918	38.25701396
rs11229433	A	G	A	G	0.0223167	-0.02200024	0.192308	NA	0.417	0.0272	1006656	3.31E-14	0.00294198	321672	0.00017885	57.54105474
rs113931125	A	G	A	G	0.0133375	0.017299497	0.383681	NA	0.382	0.0198	1006656	2.27E-08	0.00238587	321672	9.71E-05	31.25018638
rs114497064	G	C	G	C	0.0262547	-0.262102692	0.0676099	0.15	0.0595	0.1391	1006656	1.49E-08	0.00463663	321672	9.97E-05	32.06317596
rs11545169	T	G	T	G	-0.021077	-0.031604193	0.190925	NA	0.248	0.0273	1006656	9.93E-13	0.00295549	321672	0.00015808	50.85760476
rs11601751	T	C	T	C	0.0194183	0.009603736	0.500439	NA	0.624	0.0196	1006656	5.30E-17	0.00231729	321672	0.00021825	70.2196948
rs11602411	A	G	A	G	-0.0197502	0.001099395	0.215868	NA	0.968	0.0274	1006656	2.46E-12	0.00281917	321672	0.000152553	49.07923277
rs11632852	C	T	C	T	0.0134144	0.014200349	0.493032	NA	0.468	0.0196	1006656	6.66E-09	0.00231313	321672	0.00010454	33.63099543
rs11640942	T	C	T	C	-0.0401737	-0.023698607	0.125364	NA	0.436	0.0305	1006656	2.08E-30	0.0035054	321672	0.000408148	131.3426575
rs11649885	G	A	G	A	-0.0127638	-0.015702644	0.566947	NA	0.421	0.0195	1006656	4.85E-08	0.0023391	321672	9.26E-05	29.77554387
rs116681793	A	G	A	G	-0.0332094	-0.007195828	0.0417894	NA	0.919	0.0704	1006656	1.63E-08	0.0058807	321672	9.91E-05	31.89049855
rs11671304	T	C	T	C	0.0209544	0.007296555	0.632872	NA	0.726	0.0208	1006656	2.77E-18	0.0024029	321672	0.000236354	76.04599818
rs116899962	T	C	T	C	0.0458364	-0.110797491	0.0266504	NA	0.102	0.0678	1006656	2.14E-10	0.00721758	321672	0.000125363	40.33063766
rs11694327	C	T	C	T	0.0153845	0.009603736	0.279609	NA	0.683	0.0235	1006656	3.06E-09	0.00259517	321672	0.000109238	35.14248335
rs1171148	C	T	C	T	-0.0147469	-0.010201862	0.325541	NA	0.604	0.0197	1006656	2.70E-09	0.00247887	321672	0.00011001	35.39087231
rs11738695	A	C	A	C	0.0146978	0.005002492	0.578926	NA	0.792	0.0191	1006656	3.68E-10	0.00234531	321672	0.000122078	39.27366723
rs117664796	T	G	T	G	0.0249402	0.048304332	0.0850525	NA	0.092	0.0287	1006656	1.98E-09	0.00415728	321672	0.000111872	35.9897319
rs11830128	A	G	A	G	-0.0223213	-0.01640381	0.10522	NA	0.656	0.0369	1006656	3.32E-09	0.00377379	321672	0.000108748	34.98491395
rs1184523	T	G	T	G	0.0171259	-0.010801454	0.575429	NA	0.603	0.0208	1006656	3.48E-13	0.00235423	321672	0.000164484	52.91835411
rs11907606	T	C	T	C	0.0194656	0.014297305	0.201946	NA	0.624	0.0291	1006656	1.80E-11	0.00289612	321672	0.00014042	45.17516877
rs11915638	G	A	G	A	0.0249349	0.006001952	0.183934	NA	0.798	0.0234	1006656	7.62E-17	0.00299089	321672	0.000216027	69.50430101
rs11959989	A	G	A	G	0.0134556	-0.015997277	0.448447	NA	0.43	0.0202	1006656	8.08E-09	0.00233326	321672	0.000103376	33.25654774
rs12001662	T	C	T	C	-0.0162664	-0.00020002	0.496448	NA	0.992	0.0197	1006656	2.39E-12	0.00232067	321672	0.000152713	49.13075284
rs12136024	T	C	T	C	0.0205244	-0.018204705	0.128113	NA	0.455	0.0244	1006656	3.64E-09	0.00347883	321672	0.000108197	34.80741944
rs12140153	T	G	T	G	-0.0429788	-0.025399863	0.078569	NA	0.421	0.0316	1006656	3.86E-23	0.00433799	321672	0.00030506	98.1586954
rs12184680	G	A	G	A	0.0154778	-0.001901807	0.233023	NA	0.937	0.0236	1006656	1.74E-08	0.00274618	321672	9.87E-05	31.76565106
rs12264040	T	C	T	C	-0.0126341	0.033701572	0.523193	NA	0.0857	0.0196	1006656	4.89E-08	0.00231593	321672	9.25E-05	29.76014637
rs12273461	C	T	C	T	-0.0146683	-0.026600682	0.353495	NA	0.187	0.0202	1006656	1.62E-09	0.00243173	321672	0.000113101	36.3853169
rs12281369	G	A	G	A	-0.0151582	-0.006501086	0.264101	NA	0.776	0.023	1006656	8.73E-09	0.00263452	321672	0.000102904	33.10469308
rs12476198	C	T	C	T	0.0637896	0.001100605	0.834505	NA	0.967	0.0265	1006656	1.66E-92	0.00312687	321672	0.001292127	416.1763586
rs12513212	C	A	C	A	0.0127732	0.012501529	0.496198	NA	0.527	0.0198	1006656	3.64E-08	0.00231924	321672	9.43E-05	30.33229974
rs12518987	G	A	G	A	0.0139809	-0.008697716	0.326193	NA	0.663	0.02	1006656	1.54E-08	0.00247136	321672	9.95E-05	32.00335671
rs12541615	C	T	C	T	0.018738	0.002696362	0.282469	NA	0.915	0.025	1006656	3.33E-13	0.00257378	321672	0.000164747	53.00305782
rs12599980	T	C	T	C	-0.0132011	-0.042698755	0.380195	NA	0.0309	0.0198	1006656	3.48E-08	0.0023936	321672	9.46E-05	30.41686073
rs12635614	A	G	A	G	0.0154249	-0.020204507	0.553936	NA	0.302	0.0196	1006656	3.67E-11	0.00233121	321672	0.000136085	43.78034901
rs12680237	T	C	T	C	0.0135778	0.021399391	0.504767	NA	0.279	0.0197	1006656	4.49E-09	0.00231504	321672	0.000106926	34.39846917
rs12756665	T	C	T	C	-0.0139594	-0.014200349	0.443965	NA	0.469	0.0197	1006656	2.25E-09	0.00233486	321672	0.000111109	35.7444907
rs12779865	C	T	C	T	0.0157137	0.001399021	0.31307	NA	0.945	0.0211	1006656	3.22E-10	0.00249905	321672	0.000122897	39.53705565
rs12858868	G	A	G	A	-0.013022	0.012001734	0.556854	NA	0.545	0.0198	1006656	2.55E-08	0.00233807	321672	9.64E-05	31.01970497
rs12887636	G	T	G	T	-0.0235024	-0.031304926	0.397602	NA	0.127	0.0205	1006656	3.33E-23	0.00236862	321672	0.000305976	98.45345077
rs13052235	G	A	G	A	-0.0185355	0.032699496	0.500539	NA	0.0981	0.0198	1006656	1.20E-15	0.00231557	321672	0.000199156	64.07521655
rs13100903	T	C	T	C	0.0237491	0.003902376	0.282111	NA	0.854	0.0211	1006656	4.67E-20	0.0025895	321672	0.000261418	84.11235282
rs13126805	C	T	C	T	0.0160845	0.02350166	0.247277	NA	0.272	0.0214	1006656	2.18E-09	0.00268797	321672	0.000111302	35.80664209
rs13130484	T	C	T	C	0.0351043	-0.02899636	0.472039	NA	0.138	0.0195	1006656	1.89E-51	0.00232636	321672	0.000707369	227.7005558
rs13133196	T	C	T	C	-0.0153118	0.019802627	0.350524	NA	0.338	0.0207	1006656	2.79E-10	0.00242658	321672	0.000123765	39.81626954
rs13206405	A	C	A	C	0.0157377	-0.002102208	0.263089	NA	0.928	0.0236	1006656	2.36E-09	0.00263569	321672	0.000110824	35.65259672
rs1333010	A	G	A	G	-0.0231355	0.017797439	0.515629	NA	0.374	0.02	1006656	2.20E-23	0.00232202	321672	0.000308517	99.27117612
rs13362819	G	A	G	A	0.0147505	0.008196318	0.377118	NA	0.687	0.0204	1006656	7.55E-10	0.00239683	321672	0.000117727	37.8735766
rs13399978	G	A	G	A	0.0138096	-0.004098387	0.519341	NA	0.836	0.0197	1006656	2.94E-09	0.00232681	321672	0.000109491	35.22391748
rs1372504	A	G	A	G	0.0136715	0.030897711	0.34566	NA	0.122	0.02	1006656	2.11E-08	0.00244018	321672	9.76E-05	31.38960906
rs138389194	A	G	A	G	0.026233	-0.019101273	0.0710467	NA	0.673	0.0453	1006656	9.20E-09	0.00456636	321672	0.000102588	33.00296235
rs139492988	A	G	A	G	-0.0486127	0.050902218	0.0209306	NA	0.576	0.0909	1006656	2.37E-09	0.0081428	321672	0.000110787	35.64094821
rs143264991	A	C	A	C	-0.0140539	0.02059643	0.293773	NA	0.503	0.0307	1006656	4.36E-08	0.00256654	321672	9.32E-05	29.98437104
rs1436351	T	G	T	G	0.0176696	0.03740077	0.788704	NA	0.107	0.0232	1006656	4.74E-10	0.00283736	321672	0.000120548	38.78125438
rs144285608	C	A	C	A	0.0492349	-0.056295257	0.0219858	NA	0.568	0.0985	1006656	5.76E-10	0.00794512	321672	0.000119366	38.40099683
rs1445796	A	G	A	G	0.015197	0.009000382	0.696952	NA	0.667	0.021	1006656	1.59E-09	0.00251807	321672	0.000113219	36.42314383
rs1478364	T	C	T	C	-0.0150391	-0.017604046	0.275536	NA	0.501	0.0262	1006656	8.17E-09	0.00260873	321672	0.000103306	33.23400868
rs148965592	A	G	A	G	0.0384257	0.078302733	0.0438551	NA	0.0592	0.0415	1006656	1.28E-11	0.00567495	321672	0.00014251	45.84762005
rs1517037	T	C	T	C	-0.0155785	-0.03699599	0.271571	NA	0.137	0.0249	1006656	2.34E-09	0.00260864	321672	0.000110857	35.66319748
rs1582931	A	G	A	G	-0.020703	-0.005203515	0.512146	NA	0.79	0.0196	1006656	4.42E-19	0.00231941	321672	0.000247623	79.67256762
rs1652348	T	C	T	C	-0.0201539	-0.03509882	0.506025	NA	0.0714	0.0194	1006656	3.27E-18	0.00231614	321672	0.000235328	75.71569002
rs16952520	G	A	G	A	-0.0523814	-0.107395919	0.0546274	NA	0.106	0.0665	1006656	1.43E-24	0.00511968	321672	0.000325322	104.6805196
rs17024258	T	C	T	C	0.0714812	0.059098752	0.058749	NA	0.253	0.0518	1006656	1.19E-47	0.00492931	321672	0.000653303	210.2851992
rs17147219	T	G	T	G	0.0185672	-0.001100605	0.279963	NA	0.964	0.0233	1006656	6.32E-13	0.00258116	321672	0.000160835	51.74403337
rs17773492	G	A	G	A	-0.0153002	-0.045698448	0.275026	NA	0.0273	0.0207	1006656	4.60E-09	0.00261046	321672	0.000106783	34.35242733
rs17789218	C	T	C	T	0.0185921	0.030403106	0.2079	NA	0.202	0.0238	1006656	8.04E-11	0.00286043	321672	0.000131318	42.24657877
rs17797872	G	T	G	T	-0.0206683	-0.006501086	0.131181	NA	0.827	0.0296	1006656	1.92E-09	0.00344221	321672	0.000112066	36.05222637
rs17821500	C	T	C	T	0.0209623	0.002796087	0.102036	NA	0.919	0.0281	1006656	4.19E-08	0.0038235	321672	9.34E-05	30.05750824
rs1800188	T	C	T	C	-0.0400045	0.03290272	0.0727345	NA	0.579	0.0593	1006656	3.97E-19	0.00447591	321672	0.000248276	79.88262038
rs183228384	C	G	C	G	-0.0540057	0.347496955	0.0217111	0.13	0.342	0.3658	1006656	1.42E-11	0.00799363	321672	0.000141878	45.64449583
rs1865719	G	A	G	A	0.024828	0.013095371	0.649162	NA	0.512	0.02	1006656	1.43E-24	0.00242653	321672	0.000325355	104.6910229
rs1927861	T	C	T	C	0.0289422	-0.011404788	0.140417	NA	0.691	0.0286	1006656	5.27E-18	0.00334693	321672	0.00023241	74.77684231
rs1937450	G	T	G	T	0.0144306	-0.017397777	0.567216	NA	0.375	0.0196	1006656	7.52E-10	0.00234462	321672	0.00011775	37.88097483
rs1962166	A	G	A	G	-0.0192926	-0.007898723	0.724877	NA	0.708	0.0211	1006656	1.08E-13	0.00259635	321672	0.00017162	55.21439856
rs1982441	T	G	T	G	0.0231105	-0.009000382	0.184822	NA	0.747	0.028	1006656	8.11E-15	0.00297593	321672	0.000187447	60.3073937
rs1995667	A	G	A	G	-0.0141597	0.042800835	0.342721	NA	0.0463	0.0215	1006656	6.53E-09	0.00244025	321672	0.00010466	33.66950729
rs2011077	T	C	T	C	-0.0191917	-0.018703831	0.216879	NA	0.414	0.0229	1006656	9.66E-12	0.00281758	321672	0.000144211	46.39505356
rs2012697	C	T	C	T	0.0277525	0.020896826	0.644585	NA	0.29	0.0197	1006656	2.47E-30	0.00242469	321672	0.000407101	131.0053645
rs2039667	C	T	C	T	0.0141921	0.015895669	0.563323	NA	0.43	0.0201	1006656	1.42E-09	0.0023446	321672	0.000113892	36.63980078
rs2076587	A	G	A	G	-0.0176183	-0.020600746	0.352097	NA	0.309	0.0203	1006656	4.40E-13	0.00243252	321672	0.000163054	52.45808856
rs208015	C	T	C	T	-0.0284433	-0.04449524	0.894061	NA	0.248	0.0385	1006656	4.01E-14	0.00376198	321672	0.000177679	57.16422224
rs2112708	A	G	A	G	0.0158396	-0.014996981	0.568977	NA	0.442	0.0195	1006656	1.35E-11	0.00234215	321672	0.000142162	45.73580305
rs2150854	T	G	T	G	0.0239363	0.016798118	0.374747	NA	0.421	0.0209	1006656	1.45E-23	0.0023925	321672	0.000311073	100.0938618
rs2161768	G	A	G	A	0.0184935	-0.010100842	0.1782	NA	0.673	0.0239	1006656	1.12E-09	0.00303637	321672	0.00011531	37.0959194
rs2198954	G	A	G	A	0.0161467	-0.005002492	0.3444	NA	0.813	0.021	1006656	4.10E-11	0.00244636	321672	0.000135411	43.56363577
rs2208938	T	C	T	C	-0.0136681	-0.010603583	0.554308	NA	0.594	0.0199	1006656	4.86E-09	0.00233561	321672	0.000106453	34.2462437
rs2209073	A	G	A	G	-0.0140254	-0.011997738	0.63086	NA	0.546	0.0199	1006656	4.47E-09	0.002391	321672	0.000106958	34.40873128
rs2238691	A	G	A	G	-0.020152	-0.016200523	0.259316	NA	0.49	0.0235	1006656	2.63E-14	0.00264619	321672	0.000180261	57.99513463
rs2239647	C	A	C	A	-0.0248566	-0.008404582	0.569754	NA	0.669	0.0196	1006656	2.44E-26	0.00234084	321672	0.000350408	112.7553836
rs2253032	G	A	G	A	-0.0360264	0.146402442	0.959997	NA	0.0828	0.0844	1006656	1.04E-09	0.0059033	321672	0.000115768	37.24340028
rs2270623	T	C	T	C	-0.0156298	0.024497632	0.506526	NA	0.2	0.0191	1006656	1.57E-11	0.00231854	321672	0.000141255	45.44383305
rs2275003	G	A	G	A	-0.0135648	-0.037295785	0.534861	NA	0.0503	0.0191	1006656	4.78E-09	0.00231692	321672	0.000106548	34.2769375
rs2297792	C	T	C	T	0.0233296	-0.00639948	0.615639	NA	0.752	0.0201	1006656	1.33E-22	0.00238476	321672	0.000297429	95.70233362
rs2307111	C	T	C	T	-0.0203098	-0.029799631	0.424404	NA	0.139	0.0202	1006656	4.77E-18	0.0023456	321672	0.000233018	74.97227462
rs2331416	C	T	C	T	0.012944	0.006200736	0.495088	NA	0.757	0.0199	1006656	4.00E-08	0.00235733	321672	9.37E-05	30.15043219
rs2465054	A	G	A	G	-0.0161716	-0.047395637	0.385764	NA	0.0213	0.0206	1006656	1.28E-11	0.00238844	321672	0.000142496	45.84316667
rs2567302	A	G	A	G	-0.0173444	-0.007898723	0.728319	NA	0.72	0.0221	1006656	2.56E-11	0.00260036	321672	0.000138286	44.48861723
rs261230	G	A	G	A	0.015389	-0.022797909	0.575928	NA	0.244	0.0195	1006656	5.12E-11	0.00234329	321672	0.000134059	43.12866554
rs266311	A	G	A	G	0.0190266	0.024302293	0.214192	NA	0.273	0.0222	1006656	1.37E-11	0.00281437	321672	0.000142064	45.70432405
rs2681584	A	C	A	C	-0.0151814	-0.020400488	0.660045	NA	0.327	0.0208	1006656	5.61E-10	0.00244819	321672	0.000119528	38.45304241
rs272611	C	T	C	T	-0.0303959	-0.028594967	0.0529313	NA	0.571	0.0505	1006656	5.39E-09	0.00520953	321672	0.000105821	34.04318832
rs2818417	T	C	T	C	-0.0149307	0.012801592	0.574609	NA	0.542	0.0209	1006656	1.88E-10	0.00234358	321672	0.000126163	40.5880393
rs2820314	C	A	C	A	0.0181126	-0.012599036	0.296351	NA	0.542	0.0206	1006656	9.66E-13	0.00253844	321672	0.000158251	50.91257783
rs28529403	C	T	C	T	-0.0241834	-0.018102873	0.436834	NA	0.364	0.0199	1006656	6.44E-25	0.00234594	321672	0.000330251	106.2669504
rs2881654	A	G	A	G	0.0224011	-0.036404682	0.170673	NA	0.214	0.0293	1006656	3.65E-13	0.00308216	321672	0.000164189	52.8233122
rs28865339	G	A	G	A	0.0214979	0.006101349	0.134284	NA	0.836	0.0296	1006656	3.45E-10	0.00342485	321672	0.000122474	39.40091125
rs2920926	G	A	G	A	0.0187998	-0.022798136	0.732371	NA	0.292	0.0216	1006656	7.19E-13	0.00261992	321672	0.000160047	51.49056976
rs300780	T	C	T	C	0.0140355	-0.003496104	0.530998	NA	0.86	0.0198	1006656	1.59E-09	0.00232553	321672	0.000113227	36.42580715
rs34142155	A	C	A	C	0.0309305	-0.029995398	0.128958	NA	0.311	0.0296	1006656	4.97E-19	0.00347032	321672	0.000246896	79.43870113
rs34517439	A	C	A	C	0.02715	0.031004359	0.128623	NA	0.284	0.0289	1006656	5.37E-15	0.00347275	321672	0.000189975	61.12092633
rs34621693	C	A	C	A	0.0183322	0.02180063	0.144228	NA	0.404	0.0262	1006656	2.66E-08	0.00329576	321672	9.62E-05	30.93964427
rs34755	T	G	T	G	0.0146197	0.01380485	0.526347	NA	0.478	0.0194	1006656	2.98E-10	0.00232074	321672	0.000123355	39.68453044
rs34811474	A	G	A	G	-0.025355	-0.017400515	0.229026	NA	0.451	0.023	1006656	4.55E-20	0.00276372	321672	0.000261585	84.1659533
rs35058791	T	C	T	C	0.0272478	-0.003295424	0.146946	NA	0.922	0.0336	1006656	9.49E-17	0.00327854	321672	0.000214682	69.07155164
rs35088240	G	A	G	A	-0.0557514	0.032496231	0.0148046	NA	0.618	0.0651	1006656	7.19E-09	0.00963489	321672	0.000104078	33.48230387
rs35209276	A	G	A	G	0.0368419	0.110395817	0.0318392	NA	0.152	0.0772	1006656	2.49E-08	0.00660975	321672	9.66E-05	31.06785514
rs35221880	C	T	C	T	-0.0205363	-0.026700352	0.813145	NA	0.212	0.0214	1006656	7.01E-12	0.00299471	321672	0.00014617	47.02536273
rs35378894	C	T	C	T	-0.0275525	-0.006501086	0.0572686	NA	0.823	0.0289	1006656	3.45E-08	0.00499433	321672	9.46E-05	30.43440755
rs36144	C	A	C	A	0.0162212	-0.003304534	0.66192	NA	0.871	0.0203	1006656	3.55E-11	0.00244975	321672	0.000136286	43.84496473
rs3747973	G	A	G	A	-0.0130488	0.008495989	0.613137	NA	0.668	0.0197	1006656	4.40E-08	0.00238387	321672	9.31E-05	29.96217337
rs3757589	A	G	A	G	-0.0128313	0.009999835	0.435855	NA	0.622	0.0203	1006656	4.22E-08	0.00234095	321672	9.34E-05	30.04376403
rs3786791	T	C	T	C	-0.0150168	0.013498484	0.246248	NA	0.573	0.0239	1006656	2.17E-08	0.00268276	321672	9.74E-05	31.33202384
rs3789220	A	G	A	G	0.0275396	0.055595527	0.0831943	NA	0.218	0.0451	1006656	6.75E-11	0.00421991	321672	0.000132385	42.58985667
rs3790076	T	G	T	G	0.0143404	-0.024204689	0.559458	NA	0.217	0.0196	1006656	8.73E-10	0.00233896	321672	0.000116846	37.59016578
rs3800227	G	A	G	A	0.0230512	0.024497632	0.680932	NA	0.28	0.0226	1006656	2.00E-20	0.00248871	321672	0.00026663	85.78982693
rs3813663	G	T	G	T	-0.0137985	-0.039000737	0.477909	NA	0.0494	0.0199	1006656	2.91E-09	0.00232436	321672	0.000109546	35.24149061
rs3914898	A	C	A	C	-0.0187706	0.023002542	0.778601	NA	0.34	0.0241	1006656	2.25E-11	0.00280637	321672	0.000139057	44.73668005
rs3935190	A	G	A	G	-0.0184631	-0.020096702	0.503279	NA	0.305	0.0196	1006656	1.78E-15	0.00232077	321672	0.000196719	63.29105573
rs4107635	G	A	G	A	-0.013951	0.02740203	0.665638	NA	0.19	0.0209	1006656	1.44E-08	0.00246103	321672	9.99E-05	32.13469347
rs41314513	A	G	A	G	0.0371765	0.074597138	0.0339871	NA	0.425	0.0935	1006656	7.32E-09	0.0064282	321672	0.000103968	33.44687444
rs4237769	A	G	A	G	-0.0130679	0.0009995	0.398623	NA	0.959	0.0202	1006656	3.42E-08	0.00236802	321672	9.47E-05	30.45356728
rs4287756	G	A	G	A	-0.0227542	0.033898103	0.755544	NA	0.123	0.0219	1006656	3.98E-17	0.00270454	321672	0.000220002	70.78375924
rs4319898	G	A	G	A	0.0181654	0.029099308	0.629264	NA	0.139	0.0197	1006656	3.93E-14	0.00240179	321672	0.000177799	57.20278401
rs4335979	G	A	G	A	0.0136827	0.040301297	0.536506	NA	0.0388	0.0195	1006656	4.15E-09	0.00232781	321672	0.000107396	34.54982489
rs437564	T	C	T	C	0.0316016	0.02399969	0.42642	NA	0.224	0.0197	1006656	1.09E-40	0.00236608	321672	0.000554249	178.3842858
rs4388268	A	G	A	G	-0.0155412	0.033695864	0.217084	NA	0.17	0.0245	1006656	3.31E-08	0.00281322	321672	9.49E-05	30.51820641
rs4397868	T	C	T	C	0.0179209	-0.01530232	0.808368	NA	0.537	0.0247	1006656	1.19E-09	0.00294676	321672	0.000114965	36.98515138
rs4414033	A	G	A	G	0.0140687	-0.021996298	0.570003	NA	0.273	0.0201	1006656	2.04E-09	0.00234689	321672	0.000111702	35.93517537
rs4462854	A	G	A	G	-0.0135249	0.001498876	0.408205	NA	0.943	0.0201	1006656	9.68E-09	0.00235779	321672	0.000102282	32.9044914
rs4468007	T	C	T	C	-0.0140667	0.018001051	0.576895	NA	0.361	0.0197	1006656	1.95E-09	0.0023436	321672	0.000111984	36.02589441
rs4474776	C	T	C	T	0.0196307	-0.006299802	0.385746	NA	0.759	0.0207	1006656	1.56E-16	0.00237897	321672	0.000211635	68.09119267
rs4554194	A	G	A	G	-0.0184583	0.005395419	0.367845	NA	0.797	0.021	1006656	1.70E-14	0.00240614	321672	0.000182915	58.84897655
rs45551238	T	C	T	C	-0.0735249	0.04449524	0.048684	0.629	0.615	0.0885	1006656	5.13E-42	0.00541344	321672	0.000573139	184.4673588
rs4596023	G	A	G	A	0.0147058	0.044903228	0.738194	NA	0.0448	0.0224	1006656	2.87E-08	0.00265011	321672	9.57E-05	30.79263176
rs4663210	G	A	G	A	-0.0150592	-0.011602431	0.735635	NA	0.613	0.023	1006656	1.05E-08	0.00263164	321672	0.000101787	32.74523747
rs4671328	G	T	G	T	-0.0223586	-0.006601744	0.598989	NA	0.738	0.0196	1006656	3.03E-21	0.00236304	321672	0.000278236	89.52500754
rs4678496	T	C	T	C	-0.0152172	-0.000499875	0.599665	NA	0.979	0.0195	1006656	1.37E-10	0.00237071	321672	0.000128069	41.2012065
rs4711634	A	G	A	G	-0.0266469	-0.032099262	0.417304	NA	0.101	0.0196	1006656	1.46E-29	0.00236015	321672	0.000396122	127.4710235
rs4771122	A	G	A	G	-0.018525	0.048003973	0.65979	NA	0.038	0.0231	1006656	4.25E-14	0.00245266	321672	0.000177317	57.04781781
rs4776375	A	G	A	G	-0.028657	-0.021203208	0.161776	NA	0.371	0.0238	1006656	7.14E-20	0.00314029	321672	0.000258819	83.27588077
rs4780852	G	T	G	T	0.0169023	0.0037972	0.735133	NA	0.877	0.0246	1006656	1.21E-10	0.00262522	321672	0.000128852	41.45314665
rs4832043	C	T	C	T	-0.0152219	-0.008899483	0.510011	NA	0.646	0.0194	1006656	5.88E-11	0.00232511	321672	0.000133223	42.85959085
rs4912844	C	A	C	A	-0.0152856	-0.005504821	0.726155	NA	0.805	0.0223	1006656	4.37E-09	0.00260419	321672	0.000107093	34.45219901
rs4926775	G	A	G	A	-0.0167321	-0.001099395	0.643518	NA	0.957	0.0205	1006656	5.35E-12	0.00242635	321672	0.000147814	47.55446576
rs4932493	C	A	C	A	0.0137728	-0.016896448	0.477375	NA	0.387	0.0196	1006656	3.61E-09	0.00233398	321672	0.000108241	34.82150369
rs4954552	A	G	A	G	-0.0246954	0.015597721	0.365531	NA	0.521	0.0243	1006656	2.56E-24	0.00242704	321672	0.000321755	103.5322284
rs509325	G	T	G	T	0.0478115	-0.011596986	0.181345	NA	0.632	0.0242	1006656	6.41E-57	0.00300715	321672	0.000785236	252.7853229
rs538656	T	G	T	G	0.0521373	0.029801491	0.185057	NA	0.18	0.0222	1006656	7.84E-69	0.00297345	321672	0.000954877	307.4490061
rs55668221	A	G	A	G	-0.0153855	0.002097798	0.257634	NA	0.918	0.0207	1006656	6.24E-09	0.00264805	321672	0.000104933	33.75733602
rs56915529	T	C	T	C	-0.0160635	-0.032595508	0.290468	NA	0.129	0.0215	1006656	4.72E-10	0.00257916	321672	0.000120575	38.79011664
rs57636386	C	T	C	T	-0.0305096	-0.02880079	0.0502816	NA	0.397	0.0341	1006656	9.04E-09	0.00530794	321672	0.000102698	33.03834754
rs577993	T	C	T	C	0.0142139	0.008102739	0.543555	NA	0.687	0.0201	1006656	1.05E-09	0.00232957	321672	0.000115721	37.22822083
rs58970950	C	A	C	A	-0.0192573	0.04580476	0.182108	NA	0.155	0.0322	1006656	1.59E-10	0.00301059	321672	0.00012718	40.91521734
rs59133600	C	A	C	A	-0.027638	0.017898855	0.0639262	NA	0.708	0.0478	1006656	5.77E-09	0.00474617	321672	0.000105407	33.9096998
rs59956089	C	T	C	T	0.0161914	0.024497472	0.247821	NA	0.312	0.0243	1006656	1.76E-09	0.00269011	321672	0.000112607	36.22647071
rs60264701	C	T	C	T	0.0699128	-0.015001969	0.0108838	NA	0.86	0.085	1006656	6.07E-10	0.0112969	321672	0.00011905	38.29941344
rs6044685	G	A	G	A	0.0145415	0.014602857	0.428392	NA	0.454	0.0195	1006656	4.71E-10	0.00233471	321672	0.000120583	38.79268394
rs6068144	G	A	G	A	-0.0216514	-0.037504587	0.227797	NA	0.0918	0.0222	1006656	4.66E-15	0.00276313	321672	0.000190842	61.39975391
rs612389	C	T	C	T	-0.0201715	0.033502472	0.155724	NA	0.219	0.0272	1006656	2.79E-10	0.00319666	321672	0.00012377	39.81812407
rs61579019	G	T	G	T	0.0171197	-0.014697481	0.317482	NA	0.477	0.0207	1006656	6.53E-12	0.00249285	321672	0.000146596	47.16255297
rs61937571	A	G	A	G	-0.0391603	-0.007598798	0.0352413	NA	0.904	0.063	1006656	6.20E-10	0.00633129	321672	0.000118917	38.25647113
rs61992671	G	A	G	A	-0.015245	-0.022103899	0.509746	NA	0.265	0.0199	1006656	8.04E-11	0.00234547	321672	0.000131318	42.24666124
rs62107261	C	T	C	T	-0.10732	-0.048602182	0.0199006	NA	0.326	0.0494	1006656	5.76E-38	0.00833168	321672	0.000515536	165.9179851
rs62171412	G	A	G	A	-0.0251408	-0.002402885	0.124275	NA	0.954	0.0425	1006656	9.23E-13	0.00352036	321672	0.000158527	51.00130992
rs62174267	C	T	C	T	0.0380966	0.030703778	0.0288067	NA	0.457	0.0412	1006656	4.60E-08	0.0069697	321672	9.29E-05	29.87731499
rs62294143	T	C	T	C	0.0194662	0.020802128	0.121119	NA	0.495	0.0305	1006656	4.41E-08	0.00355651	321672	9.31E-05	29.95791565
rs62430555	A	G	A	G	0.0188288	0.017702384	0.218444	NA	0.416	0.0217	1006656	2.33E-11	0.00281709	321672	0.000138858	44.67259083
rs6416742	T	C	T	C	0.0210287	-0.032595508	0.296539	NA	0.116	0.0207	1006656	1.56E-16	0.00254849	321672	0.000211619	68.08575893
rs6419384	C	T	C	T	-0.016219	0.010197825	0.31026	NA	0.647	0.0224	1006656	9.68E-11	0.00250609	321672	0.000130192	41.88438281
rs6444144	T	C	T	C	0.01323	-0.018802127	0.638135	NA	0.514	0.0288	1006656	4.02E-08	0.00240981	321672	9.37E-05	30.14056462
rs6471478	C	T	C	T	0.0187968	-0.012303994	0.651542	NA	0.537	0.02	1006656	1.23E-14	0.00243701	321672	0.00018491	59.49089714
rs652105	T	C	T	C	0.0134965	0.02180063	0.41911	NA	0.263	0.0195	1006656	9.40E-09	0.00235078	321672	0.000102461	32.96215851
rs6695685	A	G	A	G	0.0196489	-0.021795812	0.467361	NA	0.276	0.02	1006656	3.45E-17	0.0023308	321672	0.00022088	71.06630995
rs6705250	G	A	G	A	0.0211412	-0.033805005	0.122567	NA	0.266	0.0304	1006656	2.30E-09	0.00353817	321672	0.000110979	35.7025452
rs6746570	C	A	C	A	-0.0150999	-0.025799975	0.286227	NA	0.234	0.0217	1006656	5.03E-09	0.0025828	321672	0.000106245	34.17935687
rs6749170	G	A	G	A	0.0295204	-0.029202266	0.411895	NA	0.139	0.0197	1006656	6.86E-36	0.00236038	321672	0.000486022	156.4148287
rs680497	T	C	T	C	0.0136787	-0.003295424	0.329558	NA	0.888	0.0231	1006656	3.17E-08	0.00247272	321672	9.51E-05	30.60110198
rs680559	G	A	G	A	-0.0150787	-0.021795812	0.44903	NA	0.266	0.0196	1006656	1.01E-10	0.00233229	321672	0.000129925	41.79843302
rs6806706	A	C	A	C	0.0136592	0.034301632	0.625241	NA	0.0835	0.0198	1006656	1.10E-08	0.00239034	321672	0.000101502	32.65340462
rs6816991	C	T	C	T	-0.0146126	-0.029202266	0.280639	NA	0.155	0.0206	1006656	1.62E-08	0.00258708	321672	9.92E-05	31.90307722
rs6833262	A	C	A	C	0.0226279	0.008899483	0.833064	NA	0.723	0.0251	1006656	2.92E-13	0.00310062	321672	0.000165541	53.25847335
rs6913550	C	T	C	T	0.0220961	-0.011602431	0.707989	NA	0.628	0.0239	1006656	5.92E-18	0.00255918	321672	0.000231694	74.54643634
rs6955671	T	C	T	C	-0.0222247	-0.011197078	0.628324	NA	0.573	0.0199	1006656	2.52E-20	0.0024059	321672	0.000265209	85.33240191
rs6956966	C	T	C	T	-0.0385307	-0.054204054	0.804334	NA	0.0474	0.0274	1006656	1.10E-39	0.00292274	321672	0.00053999	173.7923802
rs7072873	T	C	T	C	-0.015047	0.004098387	0.515066	NA	0.836	0.0197	1006656	7.97E-11	0.00231453	321672	0.000131372	42.26408909
rs7102454	C	T	C	T	0.0165371	-0.045604245	0.287203	NA	0.026	0.0205	1006656	1.05E-10	0.00256007	321672	0.000129702	41.7265352
rs7132908	A	G	A	G	0.0279704	-0.002302649	0.382615	NA	0.908	0.02	1006656	1.07E-31	0.00238758	321672	0.000426465	137.2393933
rs7148887	A	G	A	G	0.0137337	-0.016902038	0.404736	NA	0.388	0.0196	1006656	5.67E-09	0.00235724	321672	0.000105514	33.94413988
rs7159126	C	T	C	T	-0.0173348	-0.024702613	0.276785	NA	0.269	0.0223	1006656	2.03E-11	0.00258572	321672	0.000139701	44.94402684
rs71627581	A	G	A	G	-0.0267146	0.003204859	0.171051	NA	0.917	0.0303	1006656	6.85E-18	0.00310011	321672	0.000230797	74.25752044
rs7200737	A	G	A	G	0.0174306	0.00449986	0.250946	NA	0.842	0.0223	1006656	7.45E-11	0.00267695	321672	0.000131787	42.39761767
rs7203844	G	A	G	A	0.0194812	0.042695439	0.149491	NA	0.194	0.0329	1006656	2.28E-09	0.00325966	321672	0.000111026	35.71775083
rs7259484	G	A	G	A	-0.0162593	0.027498429	0.227963	NA	0.238	0.0233	1006656	3.91E-09	0.00276149	321672	0.00010776	34.66682264
rs72634501	C	T	C	T	0.0153936	-0.060195964	0.22128	NA	0.00903	0.0231	1006656	4.39E-08	0.00281189	321672	9.32E-05	29.96960644
rs72654628	G	A	G	A	0.0193243	-0.023698607	0.266368	NA	0.36	0.0259	1006656	1.87E-13	0.00262638	321672	0.00016827	54.13644293
rs72733275	T	G	T	G	0.0286463	-0.010403934	0.076605	NA	0.822	0.0463	1006656	6.60E-11	0.00438716	321672	0.000132525	42.6351136
rs72734458	G	A	G	A	-0.0146259	-0.01899935	0.329342	NA	0.375	0.0214	1006656	3.54E-09	0.00247708	321672	0.000108369	34.86281358
rs72742731	G	A	G	A	0.021036	-0.00080032	0.274349	NA	0.977	0.0265	1006656	5.74E-16	0.00259871	321672	0.000203662	65.52514472
rs72759983	T	C	T	C	-0.036006	0.02940345	0.0376049	NA	0.573	0.0521	1006656	3.36E-09	0.00608922	321672	0.000108684	34.9642103
rs72911214	C	T	C	T	-0.0250905	0.024604804	0.090812	NA	0.537	0.0398	1006656	5.06E-10	0.00403564	321672	0.000120151	38.65370214
rs73082646	A	G	A	G	0.023245	0.08469954	0.0837369	NA	0.0476	0.0428	1006656	2.98E-08	0.00419401	321672	9.55E-05	30.71832122
rs73152535	A	G	A	G	0.0156425	-0.012204169	0.303444	NA	0.565	0.0213	1006656	5.24E-10	0.00251818	321672	0.000119943	38.58656209
rs73581567	T	C	T	C	-0.0222236	0.040700387	0.231091	NA	0.131	0.027	1006656	1.14E-14	0.00287777	321672	0.000185363	59.6367491
rs73614566	C	T	C	T	0.0363777	0.008702027	0.12056	NA	0.757	0.028	1006656	1.71E-24	0.00356142	321672	0.000324243	104.332926
rs738140	G	A	G	A	-0.0176172	-0.017298765	0.323525	NA	0.408	0.021	1006656	1.04E-12	0.00247274	321672	0.000157774	50.75912895
rs7426145	A	G	A	G	-0.0242062	-0.03699599	0.0759484	NA	0.361	0.0405	1006656	4.26E-08	0.00441744	321672	9.33E-05	30.02681135
rs7434143	T	G	T	G	0.0173908	-0.004101577	0.684757	NA	0.842	0.0208	1006656	3.04E-12	0.00249299	321672	0.000151258	48.66260435
rs755555	T	C	T	C	0.022451	-0.017003748	0.307999	NA	0.416	0.0209	1006656	4.36E-19	0.00251481	321672	0.000247708	79.70000016
rs75634909	G	A	G	A	-0.0237629	0.004101577	0.0819434	NA	0.92	0.0406	1006656	2.18E-08	0.00424539	321672	9.74E-05	31.33004332
rs7563544	G	A	G	A	0.0134052	0.02989855	0.452453	NA	0.126	0.0196	1006656	8.54E-09	0.00232833	321672	0.000103038	33.14780898
rs7588732	A	G	A	G	-0.0133673	-0.002197584	0.543352	NA	0.911	0.0193	1006656	1.18E-08	0.00234432	321672	0.000101064	32.51254036
rs75947329	G	A	G	A	0.0486974	0.011701272	0.0210389	NA	0.876	0.075	1006656	3.68E-09	0.00825676	321672	0.000108126	34.78480126
rs7647305	C	T	C	T	0.0298999	0.030995434	0.817959	NA	0.194	0.0238	1006656	3.21E-23	0.00301225	321672	0.000306204	98.52688338
rs76569620	T	C	T	C	0.030548	-0.060100384	0.0462189	NA	0.225	0.0496	1006656	3.41E-08	0.00553509	321672	9.47E-05	30.45884914
rs76895963	G	T	G	T	0.0419681	0.199399888	0.0301208	NA	0.00328	0.0678	1006656	1.96E-09	0.00699298	321672	0.000111957	36.01731578
rs77159266	A	G	A	G	-0.022715	-0.053400777	0.125704	NA	0.0848	0.031	1006656	8.80E-11	0.00350205	321672	0.000130771	42.07054105
rs77479170	A	G	A	G	0.0157218	-0.016699779	0.489905	NA	0.391	0.0195	1006656	1.22E-11	0.00231953	321672	0.0001428	45.94114832
rs77505508	A	G	A	G	-0.06278	0.084396294	0.0210399	NA	0.191	0.0645	1006656	9.58E-15	0.00810614	321672	0.000186432	59.98072657
rs77669931	A	C	A	C	-0.0170119	-0.017095295	0.192026	NA	0.56	0.0293	1006656	9.00E-09	0.00295928	321672	0.000102725	33.04690744
rs7828742	G	A	G	A	0.0172232	0.015103486	0.530666	NA	0.452	0.0201	1006656	1.24E-13	0.00232369	321672	0.000170759	54.93746443
rs7860903	T	G	T	G	-0.0202718	-0.0009995	0.541401	NA	0.959	0.0196	1006656	3.17E-18	0.00232874	321672	0.00023552	75.77743828
rs7874346	C	T	C	T	0.0180998	-0.026898544	0.168513	NA	0.325	0.0273	1006656	5.64E-09	0.00310621	321672	0.000105542	33.95339647
rs78901918	A	G	A	G	0.0232993	0.017397777	0.117016	NA	0.687	0.0433	1006656	1.44E-10	0.00363411	321672	0.000127768	41.1042671
rs79027831	A	G	A	G	-0.0292904	-0.120203463	0.0655947	NA	0.00267	0.04	1006656	4.54E-10	0.00469825	321672	0.000120813	38.86652029
rs79585412	C	T	C	T	-0.032986	0.075404274	0.0827557	NA	0.0704	0.0417	1006656	4.01E-15	0.00419953	321672	0.000191761	61.69574785
rs80146384	G	A	G	A	-0.0175774	0.00809713	0.156992	NA	0.792	0.0309	1006656	3.57E-08	0.0031896	321672	9.44E-05	30.36925416
rs8022640	A	G	A	G	0.0146345	-0.050598799	0.32529	NA	0.0157	0.0209	1006656	3.69E-09	0.00248149	321672	0.000108111	34.77987517
rs80345943	A	C	A	C	0.0435029	0.153501898	0.0406849	NA	0.00557	0.0554	1006656	1.21E-13	0.005866	321672	0.000170948	54.99834239
rs8053333	G	T	G	T	-0.0150259	0.00079968	0.33531	NA	0.964	0.0171	1006656	1.03E-09	0.00246111	321672	0.000115866	37.27487863
rs8057911	T	C	T	C	0.0169455	-0.016902038	0.196804	NA	0.469	0.0233	1006656	6.49E-09	0.00291988	321672	0.000104694	33.68030911
rs8091425	C	T	C	T	-0.0147144	0.006598184	0.256797	NA	0.775	0.0232	1006656	3.03E-08	0.00265628	321672	9.54E-05	30.68560645
rs865637	C	T	C	T	0.0192927	0.008395141	0.610989	NA	0.686	0.0208	1006656	4.69E-16	0.00237613	321672	0.000204901	65.9239053
rs867559	G	A	G	A	0.0206026	-0.004600566	0.332917	NA	0.847	0.024	1006656	6.96E-17	0.00246805	321672	0.000216585	69.68406124
rs887508	G	A	G	A	0.016717	-0.000299955	0.634484	NA	0.992	0.0301	1006656	4.10E-12	0.00241096	321672	0.000149437	48.07662523
rs889970	A	G	A	G	-0.0142125	-0.00039992	0.595319	NA	0.986	0.0195	1006656	1.92E-09	0.00236703	321672	0.000112065	36.05211358
rs9272339	G	A	G	A	-0.0153092	-0.00449986	0.70339	NA	0.814	0.0193	1006656	1.89E-09	0.00254858	321672	0.000112162	36.08325869
rs9324397	T	C	T	C	-0.0327221	-0.011602431	0.953762	NA	0.843	0.0589	1006656	3.33E-09	0.00553236	321672	0.000108743	34.98313851
rs934935	A	G	A	G	0.014648	-0.027799975	0.688596	NA	0.162	0.0199	1006656	6.49E-09	0.002524	321672	0.000104693	33.68024649
rs9512706	C	A	C	A	0.0147377	-0.003304534	0.689528	NA	0.87	0.0205	1006656	3.82E-09	0.00250133	321672	0.000107909	34.71480576
rs9530843	C	A	C	A	-0.0170149	0.051500773	0.327123	NA	0.00837	0.0195	1006656	5.96E-12	0.00247288	321672	0.000147155	47.34237242
rs9549099	C	T	C	T	0.0183667	-0.012902885	0.32538	NA	0.558	0.022	1006656	1.12E-13	0.00247338	321672	0.000171393	55.141411
rs9563548	G	A	G	A	-0.0209766	-0.009797842	0.272336	NA	0.661	0.0224	1006656	7.45E-16	0.00260162	321672	0.000202061	65.00994044
rs9564356	A	G	A	G	-0.0179408	0.013695783	0.214767	NA	0.534	0.0221	1006656	2.09E-10	0.00282334	321672	0.000125513	40.3789059
rs9613199	T	C	T	C	0.0136818	-0.01640381	0.528607	NA	0.403	0.0196	1006656	3.61E-09	0.00231859	321672	0.000108238	34.82056856
rs9641640	C	A	C	A	0.0236058	-0.04160355	0.0785832	NA	0.344	0.044	1006656	4.05E-08	0.00430077	321672	9.36E-05	30.12605822
rs969960	G	A	G	A	-0.016772	0.005897355	0.767559	NA	0.791	0.0224	1006656	1.04E-09	0.00274784	321672	0.000115804	37.254962
rs982692	C	T	C	T	0.0172304	0.014602857	0.267095	NA	0.471	0.0203	1006656	4.92E-11	0.0026213	321672	0.000134303	43.20704488
rs9839020	C	T	C	T	0.0166072	0.017502275	0.539776	NA	0.378	0.0198	1006656	1.04E-12	0.00233101	321672	0.000157769	50.75769108
rs9847260	G	A	G	A	-0.0314128	0.040201002	0.0432806	NA	0.348	0.0428	1006656	3.96E-08	0.00571906	321672	9.38E-05	30.16902622
rs9866123	A	G	A	G	-0.0150171	-0.015895669	0.473977	NA	0.414	0.0195	1006656	1.21E-10	0.00233265	321672	0.000128826	41.44482255
rs989393	C	T	C	T	0.0142938	-0.001300846	0.299019	NA	0.951	0.0212	1006656	1.59E-08	0.00252932	321672	9.93E-05	31.93634019
rs9955276	T	C	T	C	0.024227	-0.010201862	0.17459	NA	0.709	0.0274	1006656	1.67E-15	0.00304218	321672	0.00019712	63.42007695
Summary	NA	NA	NA	NA	NA	NA	NA	NA	NA	NA	NA	NA	NA	NA	0.047964022	15432.70352

**Table 9:** Independent IVs in the causal analysis of adult BMI (IEU OPEN GWAS) and SSS BMI, body mass index; IEU OPEN GWAS, Integrative Epidemiology Unit Open Genome-Wide Association Study; IVs, instrumental variables; NA, not applicable; SNP, single nucleotide polymorphism; SSS, sick sinus syndrome

SNP	effect_allele.exposure	other_allele.exposure	effect_allele.outcome	other_allele.outcome	beta.exposure	beta.outcome	eaf.exposure	eaf.outcome	pval.outcome	se.outcome	samplesize.outcome	se.exposure	pval.exposure	samplesize.exposure	R2	F
rs10063055	T	C	T	C	0.0136775	-0.011000282	0.253365	NA	0.628	0.0228	1006656	0.00226991	1.70E-09	461460	7.87E-05	36.30732798
rs10182416	G	A	G	A	0.0130378	0.001601281	0.512223	NA	0.934	0.0195	1006656	0.00197134	3.70E-11	461460	9.48E-05	43.74049351
rs10184537	T	C	T	C	-0.016303	-0.019998647	0.339341	NA	0.328	0.0204	1006656	0.00208355	5.10E-15	461460	0.000132659	61.22451043
rs10402950	C	T	C	T	0.0135545	0.008900275	0.289347	NA	0.672	0.0211	1006656	0.00218692	5.70E-10	461460	8.32E-05	38.41486655
rs10505836	C	A	C	A	0.0184851	0.012102946	0.860012	NA	0.66	0.0276	1006656	0.00287086	1.20E-10	461460	8.98E-05	41.45889263
rs10510025	T	C	T	C	0.0175643	0.001099395	0.247013	NA	0.96	0.0229	1006656	0.00229886	2.20E-14	461460	0.000126488	58.37605777
rs1064213	A	G	A	G	0.0149301	-0.025902594	0.478377	NA	0.186	0.0196	1006656	0.00197196	3.70E-14	461460	0.000124206	57.32279372
rs10742752	C	T	C	T	0.0117979	0.0010005	0.612239	NA	0.962	0.0201	1006656	0.00202961	6.10E-09	461460	7.32E-05	33.78954558
rs10760277	T	C	T	C	0.0138734	-0.007901132	0.385049	NA	0.69	0.0199	1006656	0.00203825	1.00E-11	461460	0.000100386	46.32858458
rs10771041	T	C	T	C	0.0213233	-0.000300045	0.116102	NA	0.992	0.0321	1006656	0.0030884	5.00E-12	461460	0.000103291	47.66941962
rs10780248	A	G	A	G	-0.0121232	0.02210247	0.559454	NA	0.265	0.0198	1006656	0.00199415	1.20E-09	461460	8.01E-05	36.95872767
rs1078141	T	C	T	C	0.0142239	0.015804449	0.383867	NA	0.428	0.02	1006656	0.00205862	4.90E-12	461460	0.000103444	47.74007786
rs10799778	G	T	G	T	-0.0182237	-0.034700899	0.833718	NA	0.202	0.0272	1006656	0.00264876	6.00E-12	461460	0.000102567	47.33539591
rs10824211	T	C	T	C	0.0207688	0.014297305	0.139491	NA	0.62	0.0288	1006656	0.00286631	4.30E-13	461460	0.000113761	52.50185018
rs10927006	C	T	C	T	-0.0168156	0.02810143	0.143704	NA	0.275	0.0257	1006656	0.00281467	2.30E-09	461460	7.73E-05	35.69175354
rs10960293	T	G	T	G	-0.0140984	0.02989855	0.333719	NA	0.149	0.0207	1006656	0.00210242	2.00E-11	461460	9.74E-05	44.96750748
rs10989067	A	G	A	G	0.0169514	-0.011000282	0.31593	NA	0.603	0.0212	1006656	0.00212349	1.40E-15	461460	0.000138076	63.72484994
rs11001963	T	C	T	C	0.0116794	-0.011404718	0.581338	NA	0.565	0.0198	1006656	0.00201921	7.30E-09	461460	7.25E-05	33.45616875
rs11009685	T	C	T	C	-0.0130834	-0.026600682	0.244096	NA	0.247	0.023	1006656	0.00230766	1.40E-08	461460	6.97E-05	32.14368858
rs11012732	G	A	G	A	0.0216425	0	0.331683	NA	1	0.0046	1006656	0.00210126	7.10E-25	461460	0.000229838	106.0848541
rs11057072	G	A	G	A	0.0128422	0.031101301	0.233706	NA	0.175	0.023	1006656	0.00233829	4.00E-08	461460	6.54E-05	30.1633957
rs11079849	T	C	T	C	-0.020093	-0.028697873	0.328531	NA	0.172	0.021	1006656	0.00211163	1.80E-21	461460	0.000196171	90.54240777
rs11099020	T	C	T	C	-0.0142038	-0.053597637	0.640609	NA	0.00839	0.0203	1006656	0.00206202	5.60E-12	461460	0.000102812	47.44838863
rs11115160	A	G	A	G	-0.0130768	0.020204507	0.237797	NA	0.368	0.0225	1006656	0.00233641	2.20E-08	461460	6.79E-05	31.32585751
rs11122450	G	T	G	T	-0.0116316	0.010201862	0.611739	NA	0.612	0.0201	1006656	0.00202425	9.10E-09	461460	7.15E-05	33.01784614
rs11134679	G	A	G	A	0.0182459	-0.002297359	0.684753	NA	0.916	0.0218	1006656	0.00213286	1.20E-17	461460	0.000158563	73.18195369
rs11150745	G	A	G	A	-0.0211611	0.001898197	0.317711	NA	0.927	0.0209	1006656	0.00212961	2.90E-23	461460	0.000213919	98.73575283
rs111598585	T	C	T	C	-0.0142245	0.012995195	0.208671	NA	0.589	0.024	1006656	0.00243798	5.40E-09	461460	7.38E-05	34.04174858
rs11165643	T	C	T	C	0.0193319	-0.02889839	0.590103	NA	0.146	0.0199	1006656	0.00200337	4.90E-22	461460	0.000201746	93.11611877
rs11218510	A	G	A	G	-0.0144616	0.016001294	0.40047	NA	0.421	0.0199	1006656	0.00202121	8.40E-13	461460	0.000110925	51.19268769
rs113079574	T	C	T	C	-0.0155896	-0.031098587	0.19279	NA	0.225	0.0256	1006656	0.00251598	5.80E-10	461460	8.32E-05	38.39314535
rs113603865	T	C	T	C	0.0186013	-0.075003758	0.21205	NA	0.00144	0.0235	1006656	0.00242689	1.80E-14	461460	0.000127291	58.74684226
rs113624107	A	G	A	G	0.0150461	-0.015205013	0.22586	NA	0.504	0.0228	1006656	0.00236876	2.10E-10	461460	8.74E-05	40.34631585
rs11525873	C	T	C	T	-0.0239781	0.023804413	0.097701	NA	0.486	0.0342	1006656	0.00333603	6.60E-13	461460	0.00011194	51.66158863
rs11607476	C	A	C	A	0.015712	0.009801805	0.486574	NA	0.604	0.0188	1006656	0.00199179	3.10E-15	461460	0.000134829	62.22629784
rs11608710	G	T	G	T	0.0248489	-0.033701572	0.062119	NA	0.409	0.0408	1006656	0.00415281	2.20E-09	461460	7.76E-05	35.80373476
rs11630647	A	G	A	G	-0.012674	0.019400586	0.251739	NA	0.384	0.0223	1006656	0.00228082	2.70E-08	461460	6.69E-05	30.87759725
rs116374395	A	G	A	G	0.0318817	-0.054804715	0.035444	NA	0.229	0.0455	1006656	0.00536236	2.80E-09	461460	7.66E-05	35.34834831
rs11642090	C	T	C	T	0.0114184	0.021399391	0.373536	NA	0.29	0.0202	1006656	0.00205925	2.90E-08	461460	6.66E-05	30.74613105
rs11656076	A	G	A	G	-0.0154285	-0.002102208	0.224774	NA	0.928	0.0235	1006656	0.00237054	7.60E-11	461460	9.18E-05	42.35951233
rs1167311	A	G	A	G	-0.0192649	0.010899181	0.681309	NA	0.601	0.0209	1006656	0.00213109	1.60E-19	461460	0.000177059	81.71994269
rs11691869	A	C	A	C	-0.0193158	0.014898465	0.362038	NA	0.464	0.0204	1006656	0.00205562	5.60E-21	461460	0.000191303	88.29535332
rs11699828	A	G	A	G	-0.0335418	-0.016495303	0.035832	NA	0.754	0.0526	1006656	0.00582677	8.60E-09	461460	7.18E-05	33.13714566
rs11709402	G	A	G	A	0.0228192	0.019802627	0.278732	NA	0.352	0.0213	1006656	0.00220796	4.90E-25	461460	0.000231411	106.8111386
rs11757278	C	T	C	T	-0.0146269	-0.030098448	0.303879	NA	0.147	0.0208	1006656	0.00214834	9.90E-12	461460	0.000100443	46.35500934
rs11778219	G	A	G	A	0.015796	0.047904057	0.163123	NA	0.0678	0.0262	1006656	0.0026874	4.20E-09	461460	7.49E-05	34.54838433
rs118136827	T	G	T	G	-0.0132582	-0.021795812	0.281086	NA	0.329	0.0223	1006656	0.0022019	1.70E-09	461460	7.86E-05	36.25534707
rs1191600	A	C	A	C	-0.0122197	0.016902038	0.593587	NA	0.384	0.0194	1006656	0.00202687	1.70E-09	461460	7.88E-05	36.34690335
rs12072739	G	A	G	A	0.0157008	0.028597175	0.224478	NA	0.201	0.0224	1006656	0.00236691	3.30E-11	461460	9.53E-05	44.00258512
rs12089815	A	G	A	G	-0.0123121	-0.005295952	0.54861	NA	0.787	0.0196	1006656	0.00198606	5.70E-10	461460	8.33E-05	38.43064353
rs12140153	T	G	T	G	-0.033075	-0.025399863	0.094252	NA	0.421	0.0316	1006656	0.0034589	1.20E-21	461460	0.000198109	91.43696676
rs12149660	A	G	A	G	-0.0227338	-0.041395075	0.114956	NA	0.14	0.0281	1006656	0.00311586	3.00E-13	461460	0.000115346	53.23365869
rs12259464	A	G	A	G	0.0130895	0.019498873	0.48447	NA	0.319	0.0195	1006656	0.00198686	4.50E-11	461460	9.40E-05	43.4019957
rs12273545	T	C	T	C	0.0248393	0.018399683	0.056505	NA	0.647	0.0403	1006656	0.00428551	6.80E-09	461460	7.28E-05	33.5947797
rs1229984	C	T	C	T	0.037357	-0.02580419	0.972775	NA	0.78	0.0921	1006656	0.00599385	4.60E-10	461460	8.42E-05	38.84457392
rs12340969	T	C	T	C	-0.0208868	-0.008495989	0.441399	NA	0.663	0.0195	1006656	0.0019941	1.10E-25	461460	0.000237691	109.7104679
rs12364470	G	T	G	T	0.0192705	-0.017604046	0.164556	NA	0.495	0.0257	1006656	0.0026658	4.90E-13	461460	0.000113226	52.25513293
rs12440603	T	C	T	C	0.0139285	0.016099701	0.433693	NA	0.409	0.0195	1006656	0.0020011	3.40E-12	461460	0.000104976	48.44726121
rs12462975	A	G	A	G	0.0195822	0.017496048	0.329692	NA	0.397	0.0207	1006656	0.00212071	2.60E-20	461460	0.000184734	85.26258842
rs12541408	C	T	C	T	-0.0143261	0.022504855	0.31742	NA	0.274	0.0205	1006656	0.00212723	1.60E-11	461460	9.83E-05	45.3550005
rs1263629	G	A	G	A	0.0176064	-0.018500076	0.143824	NA	0.504	0.0276	1006656	0.00282352	4.50E-10	461460	8.43E-05	38.88279806
rs1266874	G	A	G	A	0.0140966	0.02810143	0.349619	NA	0.179	0.0209	1006656	0.0020712	1.00E-11	461460	0.000100371	46.32151936
rs12681792	A	C	A	C	0.0148693	-0.008102739	0.1926	NA	0.733	0.0236	1006656	0.00251693	3.50E-09	461460	7.56E-05	34.90092125
rs12692596	T	C	T	C	0.0130872	-0.003696825	0.37185	NA	0.854	0.0203	1006656	0.00203738	1.30E-10	461460	8.94E-05	41.26173822
rs12696039	G	A	G	A	-0.0152802	0.034401427	0.149405	NA	0.198	0.0267	1006656	0.0027705	3.50E-08	461460	6.59E-05	30.41864652
rs12712767	A	G	A	G	0.0114221	0.003404199	0.370925	NA	0.866	0.0198	1006656	0.00204487	2.30E-08	461460	6.76E-05	31.20028972
rs12881629	G	A	G	A	0.0220748	0.038595518	0.08265	NA	0.274	0.0353	1006656	0.00358929	7.70E-10	461460	8.20E-05	37.8246201
rs12921986	G	A	G	A	0.0203327	-0.100196096	0.077984	NA	0.00715	0.0373	1006656	0.0036983	3.80E-08	461460	6.55E-05	30.22622762
rs12937411	T	C	T	C	-0.017187	0.00059982	0.408167	NA	0.977	0.0202	1006656	0.00201383	1.40E-17	461460	0.000157816	72.8371022
rs1296328	C	A	C	A	-0.018862	-0.004798469	0.559032	NA	0.808	0.0197	1006656	0.001999	3.90E-21	461460	0.0001929	89.03238564
rs12974458	T	C	T	C	0.0152466	-0.013597139	0.543175	NA	0.491	0.0197	1006656	0.00199717	2.30E-14	461460	0.000126278	58.27926465
rs13012070	A	G	A	G	-0.0136501	-0.039895341	0.228388	NA	0.0833	0.023	1006656	0.00234795	6.10E-09	461460	7.32E-05	33.7980851
rs13033310	A	G	A	G	0.012611	-0.015103486	0.25276	NA	0.5	0.0224	1006656	0.0022826	3.30E-08	461460	6.61E-05	30.52372578
rs13107325	T	C	T	C	0.0475799	0.048199514	0.07492	NA	0.383	0.0553	1006656	0.00375479	8.50E-37	461460	0.000347849	160.5734958
rs13176429	C	T	C	T	0.0141555	-0.005395419	0.687609	NA	0.792	0.0207	1006656	0.00213122	3.10E-11	461460	9.56E-05	44.11557862
rs1320251	T	C	T	C	-0.0180321	0.036100466	0.454791	NA	0.0646	0.0195	1006656	0.00199407	1.50E-19	461460	0.000177174	81.7730003
rs1322842	G	A	G	A	-0.0131292	-0.033695864	0.609274	NA	0.089	0.0198	1006656	0.00203539	1.10E-10	461460	9.02E-05	41.6082426
rs13248187	C	T	C	T	0.015764	0.006796849	0.268557	NA	0.761	0.0223	1006656	0.00224155	2.00E-12	461460	0.000107166	49.45772196
rs1327259	G	A	G	A	-0.0148532	-0.047301273	0.387722	NA	0.0194	0.0203	1006656	0.00203363	2.80E-13	461460	0.000115588	53.34507078
rs13291723	A	G	A	G	0.0110318	0.045499584	0.570683	NA	0.0217	0.0198	1006656	0.00199876	3.40E-08	461460	6.60E-05	30.46278309
rs13427822	G	A	G	A	-0.0181515	-0.001300846	0.271198	NA	0.95	0.0214	1006656	0.00224142	5.60E-16	461460	0.000142096	65.58079426
rs1346841	A	G	A	G	-0.013056	0.008801156	0.405041	NA	0.666	0.0205	1006656	0.0020172	9.70E-11	461460	9.08E-05	41.89097626
rs1360201	T	C	T	C	0.0130075	0.019896632	0.481546	NA	0.307	0.0195	1006656	0.00197747	4.80E-11	461460	9.38E-05	43.26791618
rs140159717	T	C	T	C	-0.0246882	0.066199641	0.082292	NA	0.0626	0.0355	1006656	0.00370967	2.80E-11	461460	9.60E-05	44.29007501
rs1411432	C	A	C	A	0.0214668	0.043796765	0.186225	NA	0.0979	0.0265	1006656	0.0025491	3.70E-17	461460	0.00015366	70.91841031
rs1441264	A	G	A	G	0.0179033	-0.027196792	0.593681	NA	0.17	0.0198	1006656	0.00205854	3.40E-18	461460	0.000163886	75.63898201
rs1451963	T	G	T	G	0.022201	-0.005002492	0.082266	NA	0.898	0.0387	1006656	0.00360585	7.40E-10	461460	8.21E-05	37.9077387
rs1458156	T	C	T	C	0.014075	-0.02059643	0.488431	NA	0.29	0.0195	1006656	0.00197951	1.20E-12	461460	0.000109547	50.55679386
rs145981104	G	A	G	A	0.022711	-0.00940408	0.063711	NA	0.82	0.0413	1006656	0.00404552	2.00E-08	461460	6.83E-05	31.51533495
rs146569428	A	G	A	G	0.0139533	0.021203606	0.200749	NA	0.378	0.0241	1006656	0.00248571	2.00E-08	461460	6.83E-05	31.51019294
rs1471093	A	G	A	G	0.0134619	0.002904213	0.616663	NA	0.888	0.0202	1006656	0.00203996	4.10E-11	461460	9.44E-05	43.54793184
rs147568678	C	T	C	T	-0.0133327	-0.058795047	0.23807	NA	0.0121	0.0234	1006656	0.00233034	1.10E-08	461460	7.09E-05	32.73374933
rs1477290	C	T	C	T	0.0337772	-0.002302649	0.136947	NA	0.939	0.0293	1006656	0.00289815	2.20E-31	461460	0.000294268	135.8324986
rs147730268	T	G	T	G	-0.0350799	-0.0037972	0.087243	NA	0.923	0.0389	1006656	0.00358357	1.30E-22	461460	0.000207616	95.82592643
rs1503526	C	T	C	T	0.0154308	0.006300113	0.480077	NA	0.745	0.0195	1006656	0.00197681	5.90E-15	461460	0.000132025	60.93195936
rs1582931	A	G	A	G	-0.0133419	-0.005203515	0.473242	NA	0.79	0.0196	1006656	0.0019956	2.30E-11	461460	9.69E-05	44.69783517
rs1609010	G	A	G	A	0.0209773	-0.006598184	0.565728	NA	0.734	0.0196	1006656	0.0019963	7.90E-26	461460	0.000239227	110.4194762
rs16965658	G	A	G	A	-0.0243736	0.020204507	0.060091	NA	0.609	0.0395	1006656	0.00418404	5.70E-09	461460	7.35E-05	33.9348412
rs17056301	C	T	C	T	0.0135831	1.00E-04	0.256425	NA	0.992	0.0104	1006656	0.0022697	2.20E-09	461460	7.76E-05	35.81450874
rs17149254	C	T	C	T	-0.0213507	-0.01400152	0.804854	NA	0.58	0.0254	1006656	0.00255804	7.00E-17	461460	0.000150942	69.66388522
rs17289010	G	A	G	A	-0.0134661	-0.009797842	0.32787	NA	0.643	0.0212	1006656	0.00210438	1.60E-10	461460	8.87E-05	40.94807188
rs17399739	G	A	G	A	0.0270713	0.061198575	0.068893	NA	0.0755	0.0344	1006656	0.00391006	4.40E-12	461460	0.000103866	47.93464305
rs17544384	C	T	C	T	0.0140931	0.004001981	0.210864	NA	0.87	0.0243	1006656	0.00241434	5.30E-09	461460	7.38E-05	34.07331007
rs17724992	G	A	G	A	-0.017199	0.002197584	0.26777	NA	0.922	0.0227	1006656	0.00224218	1.70E-14	461460	0.00012749	58.83876682
rs17770336	T	C	T	C	0.0242931	0.017997077	0.322437	NA	0.391	0.021	1006656	0.00211161	1.30E-30	461460	0.000286734	132.3538484
rs1778830	A	G	A	G	0.0140775	0.003503854	0.362165	NA	0.861	0.0201	1006656	0.00205596	7.50E-12	461460	0.000101588	46.88348297
rs1788808	G	A	G	A	-0.020404	-0.035803359	0.49478	NA	0.0656	0.0195	1006656	0.00198255	7.70E-25	461460	0.000229482	105.9206042
rs1805123	G	T	G	T	-0.0167569	0.001598721	0.245301	NA	0.945	0.0234	1006656	0.00229711	3.00E-13	461460	0.000115303	53.21350786
rs1834144	A	C	A	C	-0.0140112	0.010999285	0.373192	NA	0.581	0.02	1006656	0.00205213	8.60E-12	461460	0.00010101	46.6164335
rs1861410	T	C	T	C	-0.0212558	-0.005295952	0.555423	NA	0.787	0.0197	1006656	0.00198918	1.20E-26	461460	0.00024738	114.1838967
rs1884897	G	A	G	A	0.020001	-0.006498837	0.627378	NA	0.747	0.0202	1006656	0.00205634	2.30E-22	461460	0.00020497	94.60447727
rs1919243	C	T	C	T	0.0116759	-0.00060018	0.487406	NA	0.975	0.0196	1006656	0.00200175	5.50E-09	461460	7.37E-05	34.02194803
rs1928706	A	G	A	G	-0.0119847	0.032502531	0.471082	NA	0.095	0.0195	1006656	0.00198846	1.70E-09	461460	7.87E-05	36.32609665
rs1934102	A	G	A	G	-0.0136682	-0.015997277	0.347583	NA	0.438	0.0206	1006656	0.00210374	8.20E-11	461460	9.15E-05	42.21206892
rs1967772	A	G	A	G	-0.0170434	-0.002402885	0.285117	NA	0.91	0.0212	1006656	0.00220377	1.00E-14	461460	0.000129595	59.81058606
rs1990662	C	A	C	A	0.0144775	0.004599406	0.192721	NA	0.86	0.0259	1006656	0.00251798	8.90E-09	461460	7.16E-05	33.05831492
rs2035936	T	G	T	G	0.0370863	0.030897711	0.055892	NA	0.491	0.0448	1006656	0.00435778	1.70E-17	461460	0.000156926	72.42600482
rs2051559	C	T	C	T	0.0204078	-0.012902885	0.13254	NA	0.649	0.0284	1006656	0.00291963	2.80E-12	461460	0.000105866	48.85790735
rs2102278	G	A	G	A	0.0118583	0.060304571	0.322486	NA	0.00383	0.0208	1006656	0.0021139	2.00E-08	461460	6.82E-05	31.46835977
rs213518	C	T	C	T	0.0157894	-0.017095295	0.145608	NA	0.542	0.0281	1006656	0.00280491	1.80E-08	461460	6.87E-05	31.68775782
rs2153740	G	A	G	A	-0.0112551	0.035695275	0.479914	NA	0.0672	0.0195	1006656	0.00199313	1.60E-08	461460	6.91E-05	31.8878752
rs215634	G	A	G	A	-0.0155223	-0.034604305	0.611879	NA	0.0875	0.0203	1006656	0.00203492	2.40E-14	461460	0.000126075	58.18560882
rs2172131	C	T	C	T	-0.0149382	0.013703465	0.578723	NA	0.491	0.0199	1006656	0.00200386	9.00E-14	461460	0.000120414	55.57249619
rs217672	C	A	C	A	0.0170155	-0.005897355	0.271741	NA	0.791	0.0222	1006656	0.00223046	2.40E-14	461460	0.000126099	58.19674167
rs2192158	G	A	G	A	-0.015012	0.006903776	0.553308	NA	0.724	0.0196	1006656	0.00198302	3.70E-14	461460	0.000124175	57.30876381
rs2216931	A	C	A	C	0.0169109	-0.004101577	0.661966	NA	0.837	0.0201	1006656	0.00208587	5.20E-16	461460	0.000142417	65.72900904
rs2234458	T	C	T	C	-0.0203841	0.039198313	0.639519	NA	0.0547	0.0204	1006656	0.00205579	3.60E-23	461460	0.00021301	98.31588701
rs2271189	A	G	A	G	-0.0163086	0.013902905	0.40272	NA	0.485	0.0199	1006656	0.00201869	6.50E-16	461460	0.000141416	65.26678442
rs2275003	G	A	G	A	-0.0119236	-0.037295785	0.520932	NA	0.0503	0.0191	1006656	0.00197803	1.70E-09	461460	7.87E-05	36.33684081
rs2289379	T	C	T	C	-0.0152765	-0.004701033	0.395648	NA	0.815	0.02	1006656	0.00202952	5.20E-14	461460	0.000122765	56.65773067
rs2307111	C	T	C	T	-0.0280042	-0.029799631	0.395025	NA	0.139	0.0202	1006656	0.00202203	1.30E-43	461460	0.000415486	191.8091274
rs2342892	G	T	G	T	-0.0126972	0.012902885	0.516212	NA	0.511	0.0196	1006656	0.00197715	1.30E-10	461460	8.94E-05	41.24153302
rs2381404	C	T	C	T	0.0139664	0.05399564	0.243837	NA	0.0162	0.0224	1006656	0.00229974	1.30E-09	461460	7.99E-05	36.88158628
rs2398861	G	A	G	A	0.0179932	-0.005002492	0.259169	NA	0.82	0.022	1006656	0.00226733	2.10E-15	461460	0.000136456	62.97750681
rs2425816	A	G	A	G	0.0122284	0.049199604	0.415111	NA	0.0126	0.0197	1006656	0.00201106	1.20E-09	461460	8.01E-05	36.97322507
rs2433733	A	G	A	G	-0.0171805	0.008395141	0.677659	NA	0.681	0.0204	1006656	0.00210907	3.80E-16	461460	0.000143778	66.3571503
rs2439823	G	A	G	A	0.0191998	0.022296735	0.545612	NA	0.261	0.0198	1006656	0.00199108	5.30E-22	461460	0.000201463	92.98525948
rs2482356	C	T	C	T	-0.0113511	-0.012599036	0.429018	NA	0.528	0.0199	1006656	0.00199506	1.30E-08	461460	7.01E-05	32.37144564
rs2512892	C	T	C	T	0.0129468	-0.007303266	0.566085	NA	0.71	0.0196	1006656	0.00199819	9.20E-11	461460	9.10E-05	41.98067658
rs252761	T	G	T	G	-0.0115046	0.00800193	0.587966	NA	0.684	0.0196	1006656	0.00201769	1.20E-08	461460	7.04E-05	32.51114623
rs2568958	A	G	A	G	0.0222928	0.015103486	0.603656	NA	0.444	0.0197	1006656	0.00201204	1.60E-28	461460	0.000265954	122.7592246
rs2569993	C	T	C	T	0.01267	0.045995787	0.320327	NA	0.0244	0.0205	1006656	0.00212202	2.40E-09	461460	7.72E-05	35.64941454
rs2606228	C	A	C	A	-0.0138791	-0.008602889	0.646368	NA	0.671	0.0201	1006656	0.00208379	2.70E-11	461460	9.61E-05	44.36217477
rs2616143	A	G	A	G	-0.0138738	-0.014798967	0.319862	NA	0.471	0.0206	1006656	0.00212547	6.70E-11	461460	9.23E-05	42.60680984
rs2678204	G	T	G	T	0.024161	-0.013896105	0.340162	NA	0.5	0.0206	1006656	0.00208158	3.80E-31	461460	0.000291865	134.7229908
rs2725371	G	A	G	A	-0.0160301	0.024098039	0.696115	NA	0.253	0.0211	1006656	0.00215845	1.10E-13	461460	0.00011951	55.15521672
rs2781668	T	C	T	C	0.0148965	0.022103899	0.165587	NA	0.38	0.0252	1006656	0.00265988	2.10E-08	461460	6.80E-05	31.36479922
rs2791643	T	C	T	C	-0.0133837	-0.010702523	0.761794	NA	0.646	0.0232	1006656	0.00231332	7.20E-09	461460	7.25E-05	33.47180086
rs28350	G	A	G	A	-0.0180335	-0.030199374	0.820684	NA	0.219	0.0245	1006656	0.00258234	2.90E-12	461460	0.00010567	48.76759304
rs28366156	C	T	C	T	-0.0264826	0.006001952	0.130595	NA	0.844	0.0307	1006656	0.00292978	1.60E-19	461460	0.000177027	81.70513267
rs2837996	C	T	C	T	0.0126647	-0.030500107	0.651408	NA	0.133	0.0203	1006656	0.00207859	1.10E-09	461460	8.04E-05	37.12361534
rs28404639	T	C	T	C	-0.0117129	0.009702775	0.366041	NA	0.638	0.0207	1006656	0.00205533	1.20E-08	461460	7.04E-05	32.4760997
rs28489620	A	G	A	G	-0.0153737	-0.01640381	0.290349	NA	0.442	0.0214	1006656	0.00220025	2.80E-12	461460	0.000105787	48.82147121
rs2861685	C	T	C	T	-0.0171274	-0.002803927	0.411984	NA	0.889	0.0201	1006656	0.00199763	1.00E-17	461460	0.000159276	73.51075711
rs28670671	C	T	C	T	-0.0124638	0.005803129	0.286024	NA	0.796	0.0223	1006656	0.00226426	3.70E-08	461460	6.57E-05	30.30026814
rs2899644	T	C	T	C	0.0149659	0.006995475	0.229995	NA	0.761	0.023	1006656	0.0023609	2.30E-10	461460	8.71E-05	40.18357866
rs2920503	T	C	T	C	-0.0140307	0.037199441	0.285413	NA	0.0744	0.0208	1006656	0.00219666	1.70E-10	461460	8.84E-05	40.79727084
rs2941452	G	A	G	A	0.0111622	-0.004798469	0.617751	NA	0.809	0.0198	1006656	0.00203237	4.00E-08	461460	6.54E-05	30.16422461
rs2962334	T	G	T	G	0.0432552	0.072999501	0.020065	NA	0.35	0.0781	1006656	0.00703547	7.80E-10	461460	8.19E-05	37.79971589
rs3212038	G	A	G	A	0.0149201	-0.01069701	0.328484	NA	0.606	0.0207	1006656	0.0021124	1.60E-12	461460	0.000108096	49.88721595
rs3213943	A	C	A	C	-0.0179406	0.034604305	0.131525	NA	0.278	0.0319	1006656	0.00288163	4.80E-10	461460	8.40E-05	38.76105693
rs329118	T	C	T	C	-0.0165734	0.022504855	0.419409	NA	0.252	0.0196	1006656	0.0020038	1.30E-16	461460	0.000148223	68.4088985
rs329651	T	G	T	G	0.0157218	-0.013902905	0.803977	NA	0.577	0.0249	1006656	0.00250202	3.30E-10	461460	8.56E-05	39.48399592
rs34045288	T	C	T	C	0.0234784	0.003404199	0.334435	NA	0.874	0.0212	1006656	0.00209371	3.50E-29	461460	0.000272428	125.7482813
rs34153025	C	T	C	T	-0.0389151	-0.030799472	0.022158	NA	0.715	0.0843	1006656	0.00678155	9.60E-09	461460	7.14E-05	32.92884388
rs34234296	A	G	A	G	-0.014947	0.010098835	0.392437	NA	0.617	0.0202	1006656	0.00204032	2.40E-13	461460	0.000116286	53.66728355
rs34481751	A	C	A	C	-0.0185029	-0.038397852	0.165329	NA	0.149	0.0266	1006656	0.00270404	7.80E-12	461460	0.000101455	46.82216571
rs34517439	A	C	A	C	0.038848	0.031004359	0.121787	NA	0.284	0.0289	1006656	0.00304983	3.60E-37	461460	0.000351479	162.2498051
rs34696181	C	T	C	T	0.0114345	-0.010403934	0.476084	NA	0.584	0.019	1006656	0.00198283	8.10E-09	461460	7.21E-05	33.25534925
rs34811474	A	G	A	G	-0.0285293	-0.017400515	0.230756	NA	0.451	0.023	1006656	0.00234269	4.10E-34	461460	0.000321277	148.3032614
rs349071	A	G	A	G	-0.0133062	0.025799975	0.500388	NA	0.176	0.0191	1006656	0.00198231	1.90E-11	461460	9.76E-05	45.05708256
rs35154326	G	A	G	A	-0.0130427	-0.01669865	0.274044	NA	0.444	0.0219	1006656	0.00223442	5.30E-09	461460	7.38E-05	34.07246136
rs35364449	T	C	T	C	0.0217069	-0.016800339	0.109739	NA	0.585	0.0307	1006656	0.00318335	9.20E-12	461460	0.000100751	46.49700155
rs35697587	A	G	A	G	-0.0164677	0.00940408	0.508121	NA	0.633	0.0197	1006656	0.00198067	9.20E-17	461460	0.000149776	69.12573526
rs35809007	A	G	A	G	-0.0171009	-0.04309541	0.363205	NA	0.0339	0.0203	1006656	0.00205627	9.10E-17	461460	0.000149858	69.16331068
rs35957544	T	G	T	G	-0.0196429	-0.023101103	0.574316	NA	0.243	0.0197	1006656	0.00200438	1.10E-22	461460	0.000208078	96.03934907
rs36007635	A	G	A	G	-0.021045	0.002197584	0.137698	NA	0.937	0.0278	1006656	0.00286788	2.20E-13	461460	0.000116679	53.84855173
rs36061954	T	C	T	C	0.0128445	0.019802627	0.398747	NA	0.319	0.0198	1006656	0.00201928	2.00E-10	461460	8.77E-05	40.4612631
rs3764625	G	T	G	T	-0.0117714	0.005796769	0.587594	NA	0.77	0.0197	1006656	0.00201523	5.20E-09	461460	7.39E-05	34.11969289
rs3766823	A	G	A	G	0.016056	-0.027802945	0.17196	NA	0.293	0.0264	1006656	0.0026141	8.10E-10	461460	8.17E-05	37.72492999
rs3784710	C	T	C	T	-0.0297067	-0.02880079	0.226602	NA	0.221	0.0235	1006656	0.00236042	2.50E-36	461460	0.000343121	158.3902218
rs3803286	G	A	G	A	-0.0186417	-0.012096541	0.666815	NA	0.557	0.0207	1006656	0.00209828	6.40E-19	461460	0.000171016	78.93002943
rs3807566	T	G	T	G	-0.0120727	-0.007095111	0.43832	NA	0.717	0.0195	1006656	0.00199583	1.50E-09	461460	7.93E-05	36.58978373
rs3814883	T	C	T	C	0.0240107	0.014996981	0.482402	NA	0.435	0.0193	1006656	0.00198424	1.00E-33	461460	0.000317212	146.4263916
rs3866805	A	C	A	C	0.0117835	-0.064495855	0.355683	NA	0.00172	0.0206	1006656	0.00206546	1.20E-08	461460	7.05E-05	32.54716401
rs3897102	T	C	T	C	0.0120838	-0.003997981	0.411157	NA	0.841	0.0198	1006656	0.00202716	2.50E-09	461460	7.70E-05	35.53277442
rs3901286	A	C	A	C	-0.0225553	0.022201708	0.15244	NA	0.418	0.0274	1006656	0.00275602	2.70E-16	461460	0.000145123	66.97775766
rs3902951	G	T	G	T	0.0141019	0.020302502	0.237151	NA	0.365	0.0224	1006656	0.00235267	2.00E-09	461460	7.79E-05	35.92786001
rs3935190	A	G	A	G	-0.0144864	-0.020096702	0.536784	NA	0.305	0.0196	1006656	0.00199701	4.00E-13	461460	0.000114019	52.62093785
rs394608	C	T	C	T	0.0186353	0.019804827	0.537697	NA	0.312	0.0195	1006656	0.00199484	9.50E-21	461460	0.000189078	87.26794696
rs40071	C	T	C	T	-0.0261666	0.01530232	0.179536	NA	0.556	0.0259	1006656	0.00258163	3.80E-24	461460	0.000222575	102.7317557
rs4017425	T	C	T	C	-0.0125817	0.021497269	0.470208	NA	0.271	0.0195	1006656	0.00198035	2.10E-10	461460	8.75E-05	40.36387602
rs4055791	T	C	T	C	-0.0178082	-0.039499952	0.416804	NA	0.0445	0.0197	1006656	0.00200801	7.40E-19	461460	0.000170412	78.65139396
rs41279738	G	T	G	T	0.0684263	0.059503994	0.025988	NA	0.266	0.0535	1006656	0.0062225	4.00E-28	461460	0.00026198	120.9245337
rs41315816	C	T	C	T	0.0254409	-0.0444954	0.058989	NA	0.33	0.0457	1006656	0.00419129	1.30E-09	461460	7.98E-05	36.84407326
rs4148155	G	A	G	A	-0.0229681	0.014602857	0.113268	NA	0.644	0.0316	1006656	0.00310659	1.40E-13	461460	0.00011844	54.66135316
rs4261944	G	T	G	T	0.0138553	-0.001300846	0.364935	NA	0.95	0.0203	1006656	0.00205652	1.60E-11	461460	9.84E-05	45.39041049
rs4284600	C	T	C	T	0.0119673	0.008495989	0.467122	NA	0.664	0.0196	1006656	0.00199506	2.00E-09	461460	7.80E-05	35.98144094
rs429343	G	A	G	A	-0.0173795	-0.01400152	0.576573	NA	0.478	0.0198	1006656	0.00199729	3.30E-18	461460	0.000164054	75.71648043
rs429358	C	T	C	T	-0.0266723	0.004897985	0.154166	NA	0.854	0.0264	1006656	0.00274373	2.40E-22	461460	0.000204746	94.50097919
rs4307239	G	A	G	A	0.0121413	-0.014098924	0.458941	NA	0.471	0.0196	1006656	0.00198816	1.00E-09	461460	8.08E-05	37.29287233
rs4456769	T	C	T	C	0.0146111	-0.002402885	0.333444	NA	0.908	0.0205	1006656	0.00210138	3.60E-12	461460	0.000104756	48.34535515
rs4477562	T	C	T	C	0.0296118	-0.007095111	0.128644	NA	0.81	0.0293	1006656	0.00298011	2.90E-23	461460	0.000213913	98.73318411
rs4482463	A	C	A	C	-0.0313175	0.046504778	0.923002	NA	0.204	0.0366	1006656	0.00370692	3.00E-17	461460	0.000154649	71.37495754
rs45486197	A	G	A	G	0.025766	-0.025297295	0.065685	NA	0.528	0.04	1006656	0.00403737	1.70E-10	461460	8.83E-05	40.7281815
rs4605363	C	A	C	A	0.0163638	0.025004759	0.341595	NA	0.223	0.0205	1006656	0.00207733	3.30E-15	461460	0.000134451	62.05195335
rs4625888	T	C	T	C	0.0117507	-0.014799939	0.557644	NA	0.449	0.0196	1006656	0.00198303	3.10E-09	461460	7.61E-05	35.11292641
rs4648450	A	C	A	C	-0.014837	-0.031397792	0.466824	NA	0.11	0.0197	1006656	0.00198775	8.40E-14	461460	0.000120721	55.71431392
rs4658403	T	C	T	C	-0.0188877	0.005897355	0.833659	NA	0.829	0.0273	1006656	0.00264997	1.00E-12	461460	0.000110076	50.80124492
rs4672338	T	C	T	C	0.0135646	0.000899595	0.336228	NA	0.963	0.0202	1006656	0.00208669	8.00E-11	461460	9.16E-05	42.25676355
rs4722398	T	C	T	C	0.0186993	-0.008102739	0.136137	NA	0.779	0.0288	1006656	0.00287545	7.90E-11	461460	9.16E-05	42.28995039
rs4764949	G	A	G	A	-0.0183924	0.007501791	0.325892	NA	0.715	0.0205	1006656	0.00211197	3.10E-18	461460	0.000164322	75.84019394
rs4790292	A	C	A	C	-0.0254509	-0.02290022	0.153693	NA	0.402	0.0273	1006656	0.00275603	2.60E-20	461460	0.000184767	85.27790557
rs4820410	G	A	G	A	-0.0177457	0.0036035	0.345317	NA	0.86	0.0201	1006656	0.00208527	1.70E-17	461460	0.000156913	72.420213
rs4832298	T	C	T	C	-0.015965	-0.040901991	0.686142	NA	0.0463	0.0205	1006656	0.00212268	5.40E-14	461460	0.000122569	56.56748984
rs4858940	C	T	C	T	0.0229281	0.060302174	0.885569	NA	0.0515	0.031	1006656	0.00309976	1.40E-13	461460	0.000118548	54.71143587
rs4876611	G	A	G	A	0.0197549	0.035202397	0.720243	NA	0.101	0.0215	1006656	0.00220502	3.30E-19	461460	0.000173906	80.26435554
rs4929923	C	T	C	T	0.0189424	-0.019096496	0.645204	NA	0.349	0.0204	1006656	0.00206436	4.50E-20	461460	0.000182426	84.19712327
rs512121	C	T	C	T	-0.0159359	0.011602431	0.192054	NA	0.646	0.0253	1006656	0.00252176	2.60E-10	461460	8.65E-05	39.93409125
rs529200	G	A	G	A	0.0168965	-0.025297308	0.527829	NA	0.196	0.0195	1006656	0.0019785	1.30E-17	461460	0.000158022	72.93223352
rs539515	C	A	C	A	0.0495291	-0.006803089	0.204942	NA	0.779	0.0242	1006656	0.0024426	2.00E-91	461460	0.000890216	411.1632451
rs55707359	G	T	G	T	0.0531292	0.164997356	0.015432	NA	0.0382	0.0796	1006656	0.00812381	6.20E-11	461460	9.27E-05	42.77058158
rs55714539	C	A	C	A	0.0175719	-0.018703831	0.343587	NA	0.364	0.0207	1006656	0.00210043	6.00E-17	461460	0.000151643	69.98728376
rs55726687	A	G	A	G	0.02483	-0.023903424	0.209709	NA	0.331	0.0246	1006656	0.00242628	1.40E-24	461460	0.000226902	104.7296664
rs55769038	A	G	A	G	0.0160961	0.018102873	0.590384	NA	0.365	0.02	1006656	0.00201096	1.20E-15	461460	0.000138816	64.06673274
rs557951	G	T	G	T	0.0140534	-0.004801509	0.312937	NA	0.816	0.0208	1006656	0.00213387	4.50E-11	461460	9.40E-05	43.37355483
rs558887	G	A	G	A	-0.0129976	-0.03699599	0.307507	NA	0.0828	0.0213	1006656	0.00214881	1.50E-09	461460	7.93E-05	36.58713643
rs559231	T	G	T	G	0.0134907	-0.001601281	0.393053	NA	0.934	0.0199	1006656	0.00203562	3.40E-11	461460	9.52E-05	43.92114657
rs56038322	A	G	A	G	0.0139276	-0.005304042	0.31062	NA	0.804	0.0213	1006656	0.00214833	9.00E-11	461460	9.11E-05	42.02896568
rs56133507	G	T	G	T	0.0137622	-0.006400439	0.196875	NA	0.787	0.0238	1006656	0.00247385	2.70E-08	461460	6.71E-05	30.94761046
rs56143236	T	C	T	C	0.0126667	-0.045300759	0.256388	NA	0.0465	0.0227	1006656	0.00226533	2.30E-08	461460	6.77E-05	31.26526647
rs56203622	C	T	C	T	0.0179769	-0.008203557	0.145529	NA	0.77	0.0281	1006656	0.00280183	1.40E-10	461460	8.92E-05	41.16652049
rs56352336	C	T	C	T	-0.016327	0.001898197	0.154894	NA	0.946	0.0276	1006656	0.00275021	2.90E-09	461460	7.64E-05	35.24351284
rs56399737	T	C	T	C	-0.0161325	-0.014301785	0.449124	NA	0.464	0.0196	1006656	0.00199646	6.40E-16	461460	0.000141477	65.29504697
rs56858768	A	G	A	G	0.0159188	0.016798118	0.296861	NA	0.419	0.0208	1006656	0.00217378	2.40E-13	461460	0.0001162	53.62750514
rs56893062	G	T	G	T	0.0125141	-0.014900463	0.303342	NA	0.472	0.0208	1006656	0.00215383	6.20E-09	461460	7.31E-05	33.75782957
rs56930105	T	C	T	C	0.0157557	0.082795837	0.139295	NA	0.00307	0.028	1006656	0.00286187	3.70E-08	461460	6.57E-05	30.30914737
rs57636386	C	T	C	T	-0.0412553	-0.02880079	0.08383	NA	0.397	0.0341	1006656	0.00358326	1.10E-30	461460	0.000287173	132.5564833
rs57989773	C	T	C	T	0.0133488	0.026495864	0.244995	NA	0.269	0.024	1006656	0.00236315	1.60E-08	461460	6.91E-05	31.90803001
rs58862095	T	C	T	C	-0.0229877	0.00079968	0.419266	NA	0.972	0.023	1006656	0.00200786	2.40E-30	461460	0.000283966	131.0757355
rs59068084	T	G	T	G	0.0110552	0.008602889	0.410232	NA	0.663	0.0198	1006656	0.00201048	3.80E-08	461460	6.55E-05	30.23652038
rs59227842	G	A	G	A	0.0229675	-0.011900531	0.31149	NA	0.573	0.0211	1006656	0.00215313	1.50E-26	461460	0.000246516	113.785013
rs594024	C	T	C	T	-0.0146771	0.000100005	0.554162	NA	1	0.0196	1006656	0.00199081	1.70E-13	461460	0.00011777	54.35243396
rs6023655	G	A	G	A	-0.0147346	-0.004698943	0.765741	NA	0.832	0.0223	1006656	0.00234997	3.60E-10	461460	8.52E-05	39.31426699
rs60764613	T	G	T	G	0.02099	-0.008395141	0.144883	NA	0.76	0.0273	1006656	0.00282688	1.10E-13	461460	0.00011946	55.13257138
rs61740466	A	G	A	G	-0.0135153	-0.015997277	0.237203	NA	0.494	0.0234	1006656	0.00231885	5.60E-09	461460	7.36E-05	33.97067572
rs61813324	T	C	T	C	0.0290259	-0.000900405	0.135728	NA	0.976	0.0286	1006656	0.00292038	2.80E-23	461460	0.000214026	98.7849481
rs61828641	A	G	A	G	0.0224585	0.012303994	0.10925	NA	0.708	0.0328	1006656	0.00315913	1.20E-12	461460	0.000109508	50.53876385
rs61871615	T	C	T	C	-0.0267159	0.033802213	0.091551	NA	0.313	0.0335	1006656	0.00359269	1.00E-13	461460	0.000119816	55.2965767
rs61903695	G	A	G	A	0.0166235	0.032999478	0.254964	NA	0.153	0.0231	1006656	0.00227115	2.50E-13	461460	0.000116083	53.57367222
rs61992671	G	A	G	A	-0.0161925	-0.022103899	0.49195	NA	0.265	0.0199	1006656	0.00206924	5.10E-15	461460	0.000132683	61.23563124
rs62007782	A	G	A	G	-0.0167007	-0.005002492	0.265091	NA	0.823	0.0224	1006656	0.00224215	9.40E-14	461460	0.000120214	55.48021559
rs62072006	C	A	C	A	0.0156492	0.03949952	0.144541	NA	0.163	0.0283	1006656	0.00282374	3.00E-08	461460	6.66E-05	30.7137601
rs62107261	C	T	C	T	-0.0911559	-0.048602182	0.048327	NA	0.326	0.0494	1006656	0.00460882	4.60E-87	461460	0.000847009	391.190402
rs62241847	G	A	G	A	-0.0124263	0.020204507	0.314443	NA	0.32	0.0203	1006656	0.00212897	5.30E-09	461460	7.38E-05	34.06769127
rs62246311	A	G	A	G	0.0207539	0.011800104	0.102364	NA	0.724	0.0333	1006656	0.00325487	1.80E-10	461460	8.81E-05	40.65652562
rs62379271	G	T	G	T	0.0117237	-0.019302502	0.578511	NA	0.328	0.0197	1006656	0.00200557	5.00E-09	461460	7.40E-05	34.17054155
rs6265	T	C	T	C	-0.0399185	-0.019896632	0.188472	NA	0.436	0.0255	1006656	0.00252719	3.30E-56	461460	0.000540386	249.5001185
rs6430068	A	G	A	G	0.0186066	0.027303835	0.108559	NA	0.34	0.0286	1006656	0.0031882	5.30E-09	461460	7.38E-05	34.05971782
rs6444950	A	G	A	G	0.0158525	0.012600282	0.237474	NA	0.583	0.0229	1006656	0.00232007	8.30E-12	461460	0.000101162	46.68651588
rs6474856	T	C	T	C	-0.0122041	-0.007601039	0.64022	NA	0.711	0.0206	1006656	0.00207048	3.80E-09	461460	7.53E-05	34.74301896
rs6531639	A	G	A	G	-0.0136291	-0.021499466	0.247548	NA	0.345	0.0227	1006656	0.00233909	5.70E-09	461460	7.36E-05	33.9499086
rs6545714	A	G	A	G	-0.0205219	0.030201509	0.601448	NA	0.136	0.0202	1006656	0.00201573	2.40E-24	461460	0.000224563	103.6498154
rs6560906	C	T	C	T	-0.012198	0.000100005	0.691874	NA	1	0.0204	1006656	0.00214136	1.20E-08	461460	7.03E-05	32.448603
rs6567160	C	T	C	T	0.0541723	0.028704068	0.232714	NA	0.198	0.0223	1006656	0.00234213	2.30E-118	461460	0.001157965	534.9715339
rs6575340	A	G	A	G	0.0207333	0.002904213	0.636038	NA	0.889	0.0208	1006656	0.00206189	8.70E-24	461460	0.000219067	101.1123017
rs66679256	T	C	T	C	0.0148864	0.019998687	0.445826	NA	0.309	0.0196	1006656	0.00198759	6.90E-14	461460	0.000121546	56.09496487
rs6669189	T	C	T	C	0.0171368	-0.024098039	0.401588	NA	0.227	0.0199	1006656	0.00201682	1.90E-17	461460	0.000156431	72.19768883
rs6669341	G	A	G	A	-0.0170287	-0.021301503	0.582679	NA	0.279	0.0197	1006656	0.0019986	1.60E-17	461460	0.000157293	72.59543977
rs6682438	C	T	C	T	0.0131586	-0.009098483	0.673081	NA	0.669	0.0212	1006656	0.00210047	3.70E-10	461460	8.50E-05	39.24501705
rs6705567	C	T	C	T	-0.0146168	0.028198654	0.37596	NA	0.157	0.02	1006656	0.00204919	9.80E-13	461460	0.000110245	50.87896536
rs6707827	G	A	G	A	0.0119437	-0.001698557	0.703627	NA	0.936	0.021	1006656	0.00217332	3.90E-08	461460	6.54E-05	30.20150165
rs6725931	T	C	T	C	0.0191046	0.013196695	0.847658	NA	0.635	0.0278	1006656	0.00274403	3.30E-12	461460	0.000105031	48.47259894
rs6744646	G	A	G	A	0.0554684	-0.00249688	0.828299	NA	0.924	0.0261	1006656	0.00261213	4.50E-100	461460	0.00097621	450.9203352
rs6752979	A	G	A	G	0.0125235	-0.025902594	0.31688	NA	0.221	0.0212	1006656	0.00211721	3.30E-09	461460	7.58E-05	34.9882068
rs67609008	C	T	C	T	0.0170863	-0.050903897	0.283614	NA	0.0234	0.0225	1006656	0.0022021	8.60E-15	461460	0.000130446	60.20327245
rs6831088	A	G	A	G	-0.0115222	0.012204169	0.640139	NA	0.549	0.0203	1006656	0.0020591	2.20E-08	461460	6.79E-05	31.31223439
rs6843852	T	C	T	C	0.0130897	0.04690267	0.507861	NA	0.0161	0.0195	1006656	0.00197607	3.50E-11	461460	9.51E-05	43.87860928
rs6909685	T	C	T	C	-0.014612	0.017004599	0.326809	NA	0.403	0.0203	1006656	0.00211352	4.70E-12	461460	0.000103568	47.79744929
rs6922607	G	A	G	A	0.0148848	-0.019294956	0.189847	NA	0.455	0.0259	1006656	0.00251479	3.20E-09	461460	7.59E-05	35.03326995
rs6938973	C	T	C	T	0.0182223	-0.001998003	0.601456	NA	0.918	0.0198	1006656	0.00201872	1.80E-19	461460	0.00017654	81.48024588
rs6950388	A	G	A	G	0.0155219	-0.022201708	0.795086	NA	0.341	0.0233	1006656	0.00244806	2.30E-10	461460	8.71E-05	40.20163946
rs6962980	C	A	C	A	-0.0159568	0.027802945	0.556019	NA	0.156	0.0196	1006656	0.00198814	1.00E-15	461460	0.000139574	64.41630283
rs698147	G	A	G	A	-0.0128512	0.006702411	0.543596	NA	0.734	0.0199	1006656	0.00198514	9.60E-11	461460	9.08E-05	41.90860472
rs6998660	G	A	G	A	0.0125456	-0.004198803	0.452592	NA	0.834	0.0199	1006656	0.00198902	2.80E-10	461460	8.62E-05	39.78347276
rs7024334	G	T	G	T	-0.0138177	-0.014100124	0.77914	NA	0.555	0.0239	1006656	0.00238338	6.70E-09	461460	7.28E-05	33.61112547
rs7027304	T	C	T	C	0.0145492	0.003696825	0.652661	NA	0.847	0.0194	1006656	0.00208703	3.10E-12	461460	0.000105303	48.59806324
rs7034554	G	A	G	A	-0.0129826	-0.005897355	0.373825	NA	0.768	0.0199	1006656	0.00204252	2.10E-10	461460	8.75E-05	40.4006949
rs7038943	C	T	C	T	-0.0140202	-0.003395759	0.33879	NA	0.869	0.0204	1006656	0.00208641	1.80E-11	461460	9.78E-05	45.15514326
rs704061	C	T	C	T	0.0146374	-0.013399372	0.45505	NA	0.496	0.0197	1006656	0.00198552	1.70E-13	461460	0.000117759	54.34723678
rs705145	A	C	A	C	0.0139021	-0.008203557	0.34518	NA	0.685	0.0203	1006656	0.0020798	2.30E-11	461460	9.68E-05	44.68026973
rs7070670	T	C	T	C	-0.0123274	-0.010403934	0.327923	NA	0.622	0.021	1006656	0.00211928	6.00E-09	461460	7.33E-05	33.83486242
rs7081254	C	T	C	T	-0.0142516	0.010801454	0.205739	NA	0.654	0.024	1006656	0.00245216	6.20E-09	461460	7.32E-05	33.77751994
rs7124681	A	C	A	C	0.0256977	-0.034105021	0.408353	NA	0.0842	0.0198	1006656	0.0020063	1.50E-37	461460	0.000355393	164.0570436
rs7132908	A	G	A	G	0.0297904	-0.002302649	0.384458	NA	0.908	0.02	1006656	0.00203363	1.40E-48	461460	0.000464807	214.5887288
rs71495038	A	G	A	G	0.0277998	-0.026302908	0.076936	NA	0.475	0.0369	1006656	0.00370969	6.70E-14	461460	0.000121681	56.15730038
rs7201895	A	G	A	G	-0.0149781	0.013202463	0.354291	NA	0.515	0.0203	1006656	0.00207999	6.00E-13	461460	0.000112359	51.85481334
rs7218014	C	T	C	T	0.0189524	0.036804335	0.197305	NA	0.129	0.0243	1006656	0.00249216	2.90E-14	461460	0.000125311	57.83286441
rs7232171	T	G	T	G	0.0123404	0.057194885	0.58263	NA	0.00383	0.0198	1006656	0.00200821	8.00E-10	461460	8.18E-05	37.7605526
rs723672	T	C	T	C	0.0111273	-0.012700309	0.43151	NA	0.508	0.0192	1006656	0.00200698	3.00E-08	461460	6.66E-05	30.73913361
rs7259070	C	T	C	T	0.021869	-0.00019998	0.596062	NA	0.992	0.0211	1006656	0.00203628	6.60E-27	461460	0.000249885	115.3402731
rs72649373	C	T	C	T	0.0178062	-0.03910472	0.143188	NA	0.166	0.0282	1006656	0.00287805	6.10E-10	461460	8.29E-05	38.27753327
rs72673947	G	A	G	A	0.0218864	0.017004599	0.107031	NA	0.584	0.0311	1006656	0.00321496	9.90E-12	461460	0.00010042	46.34422611
rs72892910	T	G	T	G	0.0387798	-0.010100842	0.172232	NA	0.701	0.0262	1006656	0.00262053	1.50E-49	461460	0.000474343	218.9933745
rs72976986	A	G	A	G	-0.0232257	0.00079968	0.190123	NA	0.974	0.0239	1006656	0.00254712	7.60E-20	461460	0.000180147	83.1451517
rs73026725	A	C	A	C	-0.022318	0.019400586	0.153534	NA	0.483	0.0277	1006656	0.00275052	4.90E-16	461460	0.000142654	65.8383669
rs73052033	C	T	C	T	-0.0303929	-0.023698607	0.18493	NA	0.341	0.0249	1006656	0.00254634	7.70E-33	461460	0.000308634	142.46547
rs7306534	A	G	A	G	-0.0112754	0.01440323	0.621712	NA	0.477	0.0203	1006656	0.00205437	4.10E-08	461460	6.53E-05	30.12344962
rs73124396	C	T	C	T	-0.0154472	-0.017604046	0.205144	NA	0.451	0.0234	1006656	0.0024548	3.10E-10	461460	8.58E-05	39.59728666
rs73142879	T	C	T	C	-0.0266936	-0.00359646	0.192297	NA	0.885	0.0245	1006656	0.00252257	3.60E-26	461460	0.000242599	111.9762609
rs73193736	G	A	G	A	-0.0177165	0.020302502	0.243934	NA	0.366	0.0225	1006656	0.00232002	2.20E-14	461460	0.000126352	58.31368913
rs7331420	A	G	A	G	-0.0143702	-0.02110107	0.285276	NA	0.327	0.0215	1006656	0.00220081	6.60E-11	461460	9.24E-05	42.63425136
rs7357754	G	A	G	A	0.0141146	0.012295278	0.500139	NA	0.531	0.0196	1006656	0.00198362	1.10E-12	461460	0.000109708	50.63121051
rs73601548	T	C	T	C	0.0177354	0.029102387	0.114527	NA	0.338	0.0304	1006656	0.00311639	1.30E-08	461460	7.02E-05	32.38743015
rs73985439	C	A	C	A	0.0136616	-0.003004509	0.307299	NA	0.889	0.0214	1006656	0.00214059	1.70E-10	461460	8.83E-05	40.73186203
rs745249	T	C	T	C	0.0176118	0.025297308	0.281945	NA	0.225	0.0209	1006656	0.00219724	1.10E-15	461460	0.000139206	64.24666881
rs74750282	C	T	C	T	-0.0196382	0.016503072	0.086623	NA	0.625	0.0338	1006656	0.00352561	2.50E-08	461460	6.72E-05	31.02651068
rs7498665	G	A	G	A	0.0268646	0.019302502	0.399659	NA	0.327	0.0197	1006656	0.00201997	2.30E-40	461460	0.000383151	176.876052
rs7516554	T	C	T	C	0.0120066	0.051301294	0.399941	NA	0.00932	0.0197	1006656	0.00201565	2.60E-09	461460	7.69E-05	35.48198898
rs7519259	A	G	A	G	0.0140028	-0.010296805	0.52836	NA	0.596	0.0195	1006656	0.00198381	1.70E-12	461460	0.000107956	49.82275507
rs75499503	T	C	T	C	-0.0180331	-0.002302649	0.220101	NA	0.916	0.0217	1006656	0.00241845	8.90E-14	461460	0.00012047	55.59870468
rs7571496	G	A	G	A	-0.0158916	0.040796394	0.260545	NA	0.0691	0.0224	1006656	0.00225454	1.80E-12	461460	0.000107656	49.68410581
rs76183894	C	T	C	T	-0.0219584	0.021095908	0.080747	NA	0.552	0.0355	1006656	0.00364437	1.70E-09	461460	7.87E-05	36.30400814
rs7683836	A	G	A	G	-0.0122469	0.011799339	0.557215	NA	0.544	0.0195	1006656	0.00199427	8.20E-10	461460	8.17E-05	37.71225908
rs7708584	G	A	G	A	-0.0159321	0.003395759	0.572344	NA	0.863	0.0197	1006656	0.0019949	1.40E-15	461460	0.000138201	63.78255385
rs7762794	G	A	G	A	0.0149077	0.037295785	0.285408	NA	0.0754	0.021	1006656	0.00218606	9.10E-12	461460	0.000100767	46.50452912
rs7774	A	C	A	C	0.014988	0.041295482	0.310467	NA	0.0455	0.0207	1006656	0.00215169	3.30E-12	461460	0.000105135	48.52059117
rs7776021	A	G	A	G	0.0123701	0.057003957	0.287599	NA	0.00782	0.0214	1006656	0.00218251	1.40E-08	461460	6.96E-05	32.12418026
rs78012460	G	A	G	A	-0.0370828	0.10499945	0.022673	NA	0.183	0.0788	1006656	0.00666929	2.70E-08	461460	6.70E-05	30.91604644
rs7802342	G	T	G	T	0.0122748	0.003304534	0.288566	NA	0.879	0.0217	1006656	0.00218145	1.80E-08	461460	6.86E-05	31.6618612
rs7805441	T	C	T	C	0.0133747	0.019704583	0.502261	NA	0.303	0.0191	1006656	0.00198884	1.80E-11	461460	9.80E-05	45.22374509
rs784257	C	T	C	T	0.0179315	0.029397915	0.812541	NA	0.273	0.0268	1006656	0.00254968	2.00E-12	461460	0.000107172	49.46067104
rs78605811	C	A	C	A	-0.032737	-0.00250313	0.05405	NA	0.959	0.0477	1006656	0.00444673	1.80E-13	461460	0.000117439	54.19938438
rs78886584	G	A	G	A	0.0116661	-0.026703383	0.491021	NA	0.177	0.0197	1006656	0.0019948	5.00E-09	461460	7.41E-05	34.20194374
rs7893571	T	G	T	G	0.0140553	-0.012096541	0.665897	NA	0.56	0.0208	1006656	0.00210152	2.30E-11	461460	9.69E-05	44.73127757
rs7924036	T	G	T	G	-0.0142836	0.0037972	0.503265	NA	0.847	0.0196	1006656	0.0019778	5.10E-13	461460	0.000113013	52.15653505
rs7925100	A	G	A	G	0.014725	0.051500773	0.396117	NA	0.01	0.02	1006656	0.0020221	3.30E-13	461460	0.0001149	53.0277825
rs7944782	G	T	G	T	0.0157606	0.028296599	0.509784	NA	0.141	0.0192	1006656	0.00198748	2.20E-15	461460	0.000136253	62.88369859
rs7947143	A	G	A	G	-0.0182782	-0.011000282	0.163483	NA	0.688	0.0274	1006656	0.00267522	8.30E-12	461460	0.000101151	46.6816247
rs7975187	G	A	G	A	0.0151361	-0.012295278	0.213905	NA	0.608	0.024	1006656	0.00241251	3.50E-10	461460	8.53E-05	39.36296916
rs79780963	T	C	T	C	0.0236803	-0.034198147	0.077428	NA	0.339	0.0358	1006656	0.00369591	1.50E-10	461460	8.90E-05	41.0515674
rs7996639	A	G	A	G	0.0145559	-0.00060018	0.449354	NA	0.974	0.0199	1006656	0.00200245	3.60E-13	461460	0.000114491	52.8387923
rs8015400	A	C	A	C	0.0213422	0.040999094	0.677097	NA	0.0484	0.0208	1006656	0.00211709	6.70E-24	461460	0.000220176	101.6243655
rs8025516	G	T	G	T	-0.0146558	-0.040901991	0.645928	NA	0.04	0.0199	1006656	0.00207578	1.70E-12	461460	0.000108013	49.84877965
rs8047587	T	G	T	G	0.0676323	0.023804413	0.439435	NA	0.226	0.0197	1006656	0.00199438	1.00E-200	461460	0.002485865	1149.980857
rs8063946	T	C	T	C	-0.0251967	-0.060397773	0.05448	NA	0.206	0.0478	1006656	0.00436586	7.90E-09	461460	7.22E-05	33.30779154
rs8076669	C	T	C	T	0.0140681	0.014200349	0.561561	NA	0.467	0.0195	1006656	0.00199601	1.80E-12	461460	0.000107638	49.67565311
rs8112818	G	A	G	A	-0.020696	0.004997492	0.400329	NA	0.801	0.0199	1006656	0.00202712	1.80E-24	461460	0.00022583	104.2346307
rs8132491	A	G	A	G	-0.015375	0.012096541	0.313023	NA	0.559	0.0208	1006656	0.00219317	2.40E-12	461460	0.000106489	49.14550176
rs815163	C	T	C	T	-0.016485	0.010302893	0.563148	NA	0.604	0.0198	1006656	0.00198582	1.00E-16	461460	0.000149314	68.91222303
rs852042	G	A	G	A	-0.0131207	0.003395759	0.758575	NA	0.885	0.0236	1006656	0.00231309	1.40E-08	461460	6.97E-05	32.17563057
rs862320	T	C	T	C	-0.0231703	0.019596724	0.40965	NA	0.338	0.0204	1006656	0.00201345	1.20E-30	461460	0.000286895	132.4279734
rs879620	T	C	T	C	0.0241136	0.00800193	0.613237	NA	0.693	0.0203	1006656	0.00203595	2.30E-32	461460	0.000303895	140.2774965
rs909892	A	G	A	G	-0.0184105	0.009197572	0.134817	NA	0.757	0.0298	1006656	0.00291255	2.60E-10	461460	8.66E-05	39.95604607
rs923994	G	A	G	A	-0.0145761	-0.024604804	0.783195	NA	0.305	0.024	1006656	0.00240219	1.30E-09	461460	7.98E-05	36.81849961
rs9294260	A	G	A	G	0.014782	0.001798382	0.476559	NA	0.924	0.019	1006656	0.00198818	1.00E-13	461460	0.000119776	55.27810063
rs9349235	T	C	T	C	0.0111809	0.019704583	0.410635	NA	0.32	0.0199	1006656	0.00200924	2.60E-08	461460	6.71E-05	30.96620704
rs935166	A	G	A	G	-0.0161066	0.031101301	0.506805	NA	0.112	0.0196	1006656	0.00197292	3.20E-16	461460	0.000144408	66.6479681
rs9366863	C	T	C	T	-0.0285083	-0.038200963	0.671833	NA	0.069	0.021	1006656	0.00210043	5.80E-42	461460	0.000399042	184.2147212
rs9461887	T	C	T	C	0.0148892	-0.006702411	0.277069	NA	0.77	0.023	1006656	0.00220206	1.40E-11	461460	9.91E-05	45.71750792
rs9463175	T	C	T	C	-0.0115	0.028704068	0.338958	NA	0.16	0.0204	1006656	0.00210115	4.40E-08	461460	6.49E-05	29.95571454
rs9478496	C	T	C	T	0.0181836	-0.026795823	0.164225	NA	0.31	0.0264	1006656	0.00267493	1.10E-11	461460	0.000100128	46.20968556
rs9515446	G	A	G	A	0.0151051	-0.00549507	0.447709	NA	0.78	0.0196	1006656	0.00199052	3.20E-14	461460	0.000124775	57.58537988
rs9522180	T	C	T	C	-0.0141095	0.007296555	0.553392	NA	0.71	0.0196	1006656	0.00199325	1.50E-12	461460	0.000108572	50.10693291
rs9571687	A	C	A	C	-0.0135388	-0.003496104	0.329363	NA	0.871	0.0214	1006656	0.0021094	1.40E-10	461460	8.93E-05	41.19462747
rs9638713	G	A	G	A	-0.0361735	-0.046597285	0.97477	NA	0.465	0.0638	1006656	0.00635399	1.20E-08	461460	7.02E-05	32.41052951
rs9673839	G	A	G	A	0.0130337	0.028801234	0.490967	NA	0.134	0.0192	1006656	0.00198783	5.50E-11	461460	9.32E-05	42.99075552
rs9674487	G	C	G	C	0.158445	-1.56882394	0.001338	0.377	0.0152	0.646	1006656	0.0286171	3.10E-08	461460	6.64E-05	30.65518583
rs9830592	A	C	A	C	0.0154849	0.012700309	0.582421	NA	0.518	0.0197	1006656	0.00200164	1.00E-14	461460	0.000129674	59.84708274
rs9839081	A	G	A	G	-0.011701	0.019704583	0.325232	NA	0.344	0.0208	1006656	0.00214045	4.60E-08	461460	6.48E-05	29.88366792
rs9843653	C	T	C	T	0.0294509	-0.019600848	0.511652	NA	0.304	0.0191	1006656	0.00197538	2.90E-50	461460	0.000481452	222.2767075
rs9876664	T	G	T	G	-0.0180478	-0.017797439	0.375389	NA	0.373	0.02	1006656	0.00204174	9.60E-19	461460	0.000169293	78.13502988
rs9888533	T	C	T	C	0.0120038	-0.001099395	0.538079	NA	0.956	0.0197	1006656	0.0020182	2.70E-09	461460	7.67E-05	35.37587705
rs9926784	C	T	C	T	-0.023818	-0.00020002	0.184576	NA	0.992	0.0267	1006656	0.00254677	8.60E-21	461460	0.000189502	87.46398262
rs9951619	G	T	G	T	0.0144231	0.012902885	0.767369	NA	0.571	0.0227	1006656	0.00235777	9.50E-10	461460	8.11E-05	37.42074866
Summary	NA	NA	NA	NA	NA	NA	NA	NA	NA	NA	NA	NA	NA	NA	0.053194807	24555.47146

**Table 10:** Independent IVs in the causal analysis of SSS and adult BMI (FinnGen) BMI, body mass index; FinnGen, Finnish Health Research Environment for Genomic Research; IVs, instrumental variables; NA, not applicable; SNP, single nucleotide polymorphism; SSS, sick sinus syndrome

SNP	effect_allele.exposure	other_allele.exposure	effect_allele.outcome	other_allele.outcome	beta.exposure	beta.outcome	eaf.exposure	eaf.outcome	pval.outcome	se.outcome	samplesize.outcome	pval.exposure	se.exposure	samplesize.exposure	R2	F
rs10149621	G	T	G	T	0.429799933	0.0141335	NA	0.0172529	0.120772	0.00910934	321672	3.45E-14	0.0567	1006656	5.71E-05	57.4600112
rs116307579	T	C	T	C	0.293698935	0.0100673	NA	0.0263378	0.166239	0.00727204	321672	2.78E-09	0.0494	1006656	3.51E-05	35.34679025
rs11662320	T	C	T	C	0.119000073	-0.00418987	NA	0.246742	0.119551	0.00269158	321672	3.68E-08	0.0216	1006656	3.02E-05	30.35191452
rs11711941	T	C	T	C	0.115103894	0.00337125	NA	0.465516	0.14772	0.0023288	321672	6.93E-09	0.0199	1006656	3.32E-05	33.45592306
rs2106261	T	C	T	C	0.149703664	0.00420746	NA	0.224326	0.129989	0.00277877	321672	9.94E-10	0.0245	1006656	3.71E-05	37.33634734
rs551862772	A	G	A	G	0.787101897	-0.012287	0.863	0.00298336	0.578393	0.0221095	321672	9.92E-27	0.0735	1006656	0.000113909	114.6796548
rs7689774	T	G	T	G	0.188303628	-0.00179769	NA	0.236627	0.508591	0.00271953	321672	2.26E-15	0.0238	1006656	6.22E-05	62.59830851
Summary	NA	NA	NA	NA	NA	NA	NA	NA	NA	NA	NA	NA	NA	NA	0.00036875	371.2289497

**Table 11:** Independent IVs in the causal analysis of SSS and adult BMI (IEU OPEN GWAS) BMI, body mass index; IEU OPEN GWAS, Integrative Epidemiology Unit Open Genome-Wide Association Study; IVs, instrumental variables; NA, not applicable; SNP, single nucleotide polymorphism; SSS, sick sinus syndrome

SNP	effect_allele.exposure	other_allele.exposure	effect_allele.outcome	other_allele.outcome	beta.exposure	beta.outcome	eaf.exposure	eaf.outcome	se.outcome	pval.outcome	samplesize.outcome	pval.exposure	se.exposure	samplesize.exposure	R2	F
rs10149621	G	T	G	T	0.429799933	-0.000834236	NA	0.02829	0.00630369	0.889999986	461460	3.45E-14	0.0567	1006656	5.71E-05	57.46001123
rs116307579	T	C	T	C	0.293698935	5.43E-05	NA	0.029299	0.00588997	0.98999999	461460	2.78E-09	0.0494	1006656	3.51E-05	35.34679019
rs11662320	T	C	T	C	0.119000073	0.00196918	NA	0.264916	0.00225052	0.380000353	461460	3.68E-08	0.0216	1006656	3.02E-05	30.35191429
rs2106261	T	C	T	C	0.149703664	-0.00414536	NA	0.167612	0.00264264	0.119999932	461460	9.94E-10	0.0245	1006656	3.71E-05	37.33634715
rs35813871	A	G	A	G	0.122898817	-0.00182352	NA	0.242476	0.00229329	0.429999549	461460	2.64E-08	0.0221	1006656	3.07E-05	30.92502019
rs551862772	A	G	A	G	0.787101897	-0.0150544	0.863	0.008463	0.0114767	0.190000175	461460	9.92E-27	0.0735	1006656	0.000113909	114.6796548
rs7689774	T	G	T	G	0.188303628	0.00127801	NA	0.196754	0.00248707	0.610000232	461460	2.26E-15	0.0238	1006656	6.22E-05	62.59830882
Summary	NA	NA	NA	NA	NA	NA	NA	NA	NA	NA	NA	NA	NA	NA	0.000366236	368.6980466

MVMR and meta-analysis

The MVMR analysis findings demonstrated independent effects of adult BMI on SSS after correction of TL. Analysis of the Finngen BMI data in relation to SSS indicated a causal inference supporting the effect of increased adult BMI on SSS risk. Owing to the presence of heterogeneity (P Cochran’s Q = 0.02), we used the random-effects IVW results for causal inference (OR = 1.22, 95% CI 1.04-1.44, P = 0.016). A consistent causal inference was noted in the IEU OPEN GWAS BMI data (OR = 1.19, 95% CI 1.04-1.36, P = 0.011).

**Figure 5:**
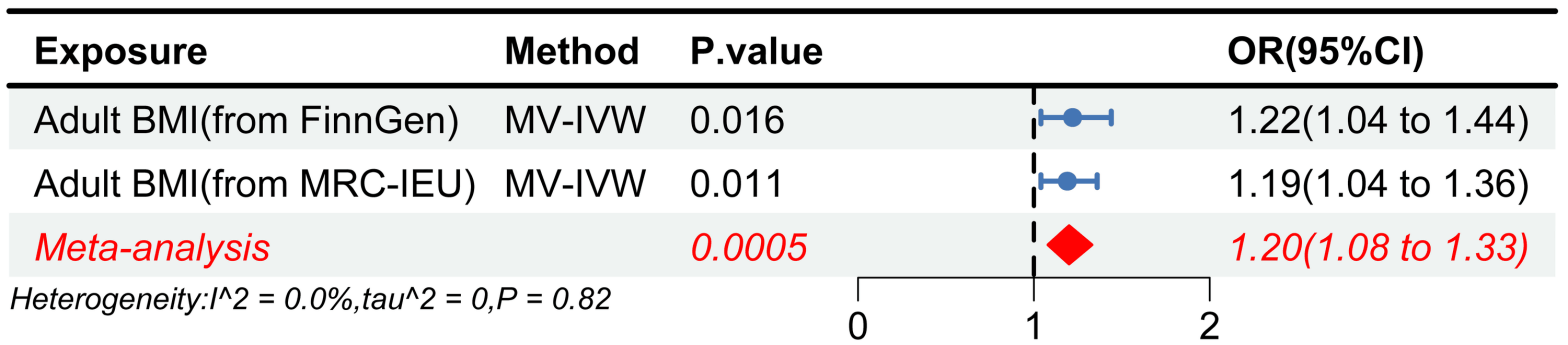
Forest plot of the MVMR causal estimates for adult BMI and SSS BMI, body mass index; CI, confidence interval; FinnGen, Finnish Health Research Environment for Genomic Research; IVW, inverse-variance weighted; MRC-IEU, Medical Research Council Integrated Epidemiology Unit; MVMR,multivariate Mendelian randomization; OR, odds ratio; SSS, sick sinus syndrome

The results of the meta-analysis supported the causality between adult BMI and SSS (OR = 1.20, 95% CI 1.08-1.33, P = 0.0005). The results of MVMR and the meta-analysis are shown in Table 12 and Figure 5, respectively.

**Table 12:** MVMR results CI, confidence interval; FinnGen, Finnish Health Research Environment for Genomic Research; IEU OPEN GWAS, Integrative Epidemiology Unit Open Genome-Wide Association Study; IVW, inverse variance weighted; MR, Mendelian randomization; OR, odds ratio

Exposure sources	Methods	nSNP	OR (95%CI)	P-value
FinnGen	MV-IVW	300	1.22 (1.04-1.44)	0.016
	MV- median		1.37 (1.09-1.71)	0.006
	MV- Egger		1.29 (0.95-1.76)	0.099
Cochrane’s Q = 352.4 (P = 0.02); MR-Egger intercept (P = 0.66); F-statistic = 41.3				
IEU OPEN GWAS	MV-IVW	368	1.19 (1.04-1.36)	0.011
	MV-median		1.04 (0.80-1.35)	0.795
	MV-Egger		1.40 (1.06-1.85)	0.017
Cochrane’s Q = 391.5(P = 0.17); MR-Egger intercept (P = 0.18); F-statistic = 55.6				

## Discussion

Evidence generated from published epidemiological studies has highlighted the association of BMI with SSS, which contradicts the findings of Thorolfsdottir et al. who used the polygenic score to investigate the relationship between BMI and SSS [4,6]. This uncertainty in association and causality paves an opportunity for an MR study. Our study findings suggest a positive association of adult BMI with SSS, and the results of replication and meta-analysis further confirmed the causal inference that the risk of SSS is increased with adult BMI and that this effect is independent of reverse causality. Given the large median age of the adult BMI population enrolled in the study, the results suggest that the risk of SSS increases with an increase in BMI among the middle-aged and older populations.

The Centers for Disease Control and Prevention predicts that children or adolescents with high BMI percentiles on the BMI growth charts are at an increased risk of becoming overweight or obese as they age [36]. Although higher BMI during childhood was not associated with an increased risk of SSS in our study, the persistence of obesity in this population may lead to a higher risk of developing SSS as they grow older. The statistical power of our study to detect associations between childhood BMI and SSS was limited, which implies a reduced likelihood of identifying true effects. This limitation may be related to smaller sample sizes and effect sizes [37]. Although we incorporated studies from diverse sources to enhance the robustness of causal inferences, future GWAS studies with larger sample sizes are necessary to validate our findings.

BMI is a simple measure of obesity. In economically developed regions, the highest prevalence of obesity is observed among individuals aged 60-69, while the upward trend in BMI among children and adolescents remains at a notably high level [38,39]. In patients with permanent pacemaker implantation, obesity is known to elevate cardiac mortality and reduce the opportunity for patients with SSS to receive optimal pacemaker therapy [40,41]. The total cost of permanent pacemaker implantation has already surged, and the growing obese population and the increase in the incidence of SSS will pose significant pressure on the healthcare economy [42].

The sinoatrial node (SN) is a heterogeneous pacemaker comprising a specialized population of cardiomyocytes. These clusters of cardiomyocytes generate action potentials spontaneously to maintain cardiac contraction [43,44]. A previous Polish study on aging and obesity in SN found that the SN of obese patients was enriched with connective tissue content, had reduced myocyte content, and showed alterations in cellular morphology as compared with the SN of non-obese patients. This observation implies that elevated SN fibrosis, apoptosis, and cellular hypertrophy in obese patients can contribute to the development of SSS. Microstructural manifestations of young obese right atrial (RA) muscle are more noteworthy than those of aging RA fibrosis [45]. In addition, the increased fat content can stimulate the release of inflammatory cytokines, which affect the SN via paracrine effects [45-47]. Studies in aged obese rats have reported SN cell hypertrophy and extracellular matrix remodeling, which are suggestive of the interaction between obesity and age that can cause SN dysfunction [45,48].

As the positive influence of weight loss on cardiovascular diseases such as heart failure and atrial fibrillation has been well documented [49-51], we anticipate that modulation of BMI could be effective for the prevention and treatment of SSS. However, there are no observational clinical studies to support this hypothesis, which may be limited by reasons such as time or costly expenses [52]. The present study offers several advantages as follows: (1) This, to the best of our knowledge, is the first comprehensive study to address the causalities between childhood and adult BMI and SSS through MR analysis. (2) The causal inference derived with SSS using a large, independent, high-quality BMI GWAS study may enhance the accuracy of the results. (3) Different methods were employed to eliminate confounders. The direction of the consistent effect estimates indicates that the causal inference findings are not by chance and that the use of reverse MR reduces the error in the causal inference [53,54]. (4) With respect to sample overlapping, which is unavoidable with the use of publicly available pooled data, the estimates were corrected using MRlap to increase the confidence of the causal inference. (5) The use of meta-analysis helped us synthesize the results of the studies, lower the possible errors in a single study, and increase the credibility of the causal interference.

The present study has some limitations: (1) There may be inconsistency in the degree of the effect of different ages on SSS, and as BMI is a continuous variable, we were unable to determine the effect of BMI on SSS at a specific age using MR. (2) To minimize bias, participants included in the MR analysis were of European descent. The causal relationships should be validated in more diverse populations to enhance the generalizability of the conclusions. (3) We cannot entirely exclude the possibility that the IVs in this study are associated with unmeasured confounders, a limitation inherent to most MR analyses. We evaluated the potential for risk factors to act as confounders in the BMI-SSS causal relationship by assessing whether they met the three key criteria for confounding. We also examined the associations between IVs and potential confounders using the Phenoscanner database. Although we cannot confirm that IVs are entirely independent of unmeasured confounders, this possibility is likely small. Additionally, the results from MVMR analyses strengthen confidence in our conclusions. (4) The potential for horizontal pleiotropy cannot be entirely excluded in this study - a methodological challenge inherent to MR analyses. Completely eliminating pleiotropic effects remains difficult, particularly for risk factors influenced by multiple genetic variants. Notably, the consistency of causal estimates across three MR approaches (IVW, MR-Egger, and weighted median) supports the robustness of our findings. (5) We cannot rule out potential influences of limited statistical power and weak instrument bias on the findings, particularly in the context of BMI reverse causation. Some results exhibited suboptimal statistical power (<80%), which may be attributed to the small sample size or effect values. Therefore, future studies with larger samples are warranted to validate these inferences. However, any bias from weak instruments is in the direction of the null, as we are considering the analyses in the two-sample setting [55].

Our findings provide MR evidence for researchers exploring the causal relationship between BMI and SSS. Specifically, focusing on adult BMI (middle-aged and older individuals), future studies should investigate the biological mechanisms underlying the association between BMI and SSS or develop targeted prevention and management strategies (e.g., weight management programs for middle-aged adults and obesity screening in primary care).

## Conclusions

This study demonstrates that a higher BMI is associated with an increased risk of SSS, particularly among middle-aged and older adults. In contrast, no causal relationship was identified between childhood BMI and SSS, suggesting that the influence of BMI on SSS susceptibility may manifest predominantly in later life stages. These findings highlight the necessity of targeted public health strategies to address adult obesity as a modifiable risk factor for SSS. Future research should focus on elucidating the biological pathways connecting adult BMI to SSS and evaluating the efficacy of interventions, such as weight reduction and lifestyle modifications, in attenuating SSS risk among individuals with elevated BMI.

## References

[1] Adán V, Crown LA (2003). Diagnosis and treatment of sick sinus syndrome. Am Fam Physician.

[2] Manoj P, Kim JA, Kim S, Li T, Sewani M, Chelu MG, Li N (2023). Sinus node dysfunction: current understanding and future directions. Am J Physiol Heart Circ Physiol.

[3] Haidar A, Gajjar A, Parikh RV (2025). National costs for cardiovascular-related hospitalizations and inpatient procedures in the United States, 2016 to 2021. Am J Cardiol.

[4] Jensen PN, Gronroos NN, Chen LY, Folsom AR, deFilippi C, Heckbert SR, Alonso A (2014). Incidence of and risk factors for sick sinus syndrome in the general population. J Am Coll Cardiol.

[5] Kusumoto FM, Schoenfeld MH, Barrett C (2019). 2018 ACC/AHA/HRS guideline on the evaluation and management of patients with bradycardia and cardiac conduction delay: executive summary: a report of the American College of Cardiology/American Heart Association task force on clinical practice guidelines, and the Heart Rhythm Society. J Am Coll Cardiol.

[6] Thorolfsdottir RB, Sveinbjornsson G, Aegisdottir HM (2021). Genetic insight into sick sinus syndrome. Eur Heart J.

[7] Semelka M, Gera J, Usman S (2013). Sick sinus syndrome: a review. Am Fam Physician.

[8] Chooi YC, Ding C, Magkos F (2019). The epidemiology of obesity. Metabolism.

[9] Hazarapetyan L, Zelveian P, Hayrapetyan H, Grigoryan S (2024). Possible risk factors contributing to atrial fibrillation occurrence in heart failure with mildly reduced ejection fraction. J Clin Med Res.

[10] Bai X, Wang H, Li J, Xu J, Cai P (2024). Correlation analysis of the risk of ischemic stroke with related risk factors in a health examination population. Pak J Med Sci.

[11] Lim CG, Ozkan B, Liang Y (2025). Plasma proteomic signatures of adiposity are associated with cardiovascular risk factors and type 2 diabetes risk in a multiethnic Asian population. Diabetes.

[12] Zhou XD, Chen QF, Yang W (2024). Erratum: burden of disease attributable to high body mass index: an analysis of data from the Global Burden of Disease study 2021. EClinicalMedicine.

[13] Davey Smith G, Hemani G (2014). Mendelian randomization: genetic anchors for causal inference in epidemiological studies. Hum Mol Genet.

[14] Burgess S, Davey Smith G, Davies NM (2019). Guidelines for performing Mendelian randomization investigations: update for summer 2023. Wellcome Open Res.

[15] Skrivankova VW, Richmond RC, Woolf BA (2021). Strengthening the reporting of observational studies in epidemiology using Mendelian randomization: the STROBE-MR statement. JAMA.

[16] Vogelezang S, Bradfield JP, Ahluwalia TS (2020). Novel loci for childhood body mass index and shared heritability with adult cardiometabolic traits. PLoS Genet.

[17] Felix JF, Bradfield JP, Monnereau C (2016). Genome-wide association analysis identifies three new susceptibility loci for childhood body mass index. Hum Mol Genet.

[18] Kurki MI, Karjalainen J, Palta P (2023). FinnGen provides genetic insights from a well-phenotyped isolated population. Nature.

[19] Sudlow C, Gallacher J, Allen N (2015). UK biobank: an open access resource for identifying the causes of a wide range of complex diseases of middle and old age. PLoS Med.

[20] Eppard M, Passos JF, Victorelli S (2024). Telomeres, cellular senescence, and aging: past and future. Biogerontology.

[21] Grover S,  Del Greco MF,  Stein CM,  Ziegler A (2017). Mendelian randomization. Statistical Human Genetics. Methods in Molecular Biology.

[22] Huang X, Yang P, Yang Z, Zhang H, Ma A (2016). Age-associated expression of HCN channel isoforms in rat sinoatrial node. Exp Biol Med (Maywood).

[23] Hao X, Zhang Y, Zhang X (2011). TGF-β1-mediated fibrosis and ion channel remodeling are key mechanisms in producing the sinus node dysfunction associated with SCN5A deficiency and aging. Circ Arrhythm Electrophysiol.

[24] Monfredi O, Boyett MR (2015). Sick sinus syndrome and atrial fibrillation in older persons - a view from the sinoatrial nodal myocyte. J Mol Cell Cardiol.

[25] Yan Z, Pu X, Chang X, Liu Z, Liu R (2025). Genetic basis and causal relationship between atrial fibrillation and sinus node dysfunction: evidence from comprehensive genetic analysis. Int J Cardiol.

[26] Kamat MA, Blackshaw JA, Young R (2019). PhenoScanner V2: an expanded tool for searching human genotype-phenotype associations. Bioinformatics.

[27] Burgess S, Thompson SG (2011). Avoiding bias from weak instruments in Mendelian randomization studies. Int J Epidemiol.

[28] Verbanck M, Chen CY, Neale B, Do R (2018). Detection of widespread horizontal pleiotropy in causal relationships inferred from Mendelian randomization between complex traits and diseases. Nat Genet.

[29] Bowden J, Davey Smith G, Haycock PC, Burgess S (2016). Consistent estimation in Mendelian randomization with some invalid instruments using a weighted median estimator. Genet Epidemiol.

[30] Vaiserman A, Krasnienkov D (2021). Telomere length as a marker of biological age: state-of-the-art, open issues, and future perspectives. Front Genet.

[31] Aubert G, Lansdorp PM (2008). Telomeres and aging. Physiol Rev.

[32] Zhu Y, Liu X, Ding X, Wang F, Geng X (2019). Telomere and its role in the aging pathways: telomere shortening, cell senescence and mitochondria dysfunction. Biogerontology.

[33] Sanders JL, Newman AB (2013). Telomere length in epidemiology: a biomarker of aging, age-related disease, both, or neither?. Epidemiol Rev.

[34] Mounier N, Kutalik Z (2023). Bias correction for inverse variance weighting Mendelian randomization. Genet Epidemiol.

[35] Deng Z, Hu Y, Duan L (2024). Causality between sleep traits and the risk of frailty: a Mendelian randomization study. Front Public Health.

[36] Guo SS, Wu W, Chumlea WC, Roche AF (2002). Predicting overweight and obesity in adulthood from body mass index values in childhood and adolescence. Am J Clin Nutr.

[37] Serdar CC, Cihan M, Yücel D, Serdar MA (2021). Sample size, power and effect size revisited: simplified and practical approaches in pre-clinical, clinical and laboratory studies. Biochem Med (Zagreb).

[38] Kelly T, Yang W, Chen CS, Reynolds K, He J (2008). Global burden of obesity in 2005 and projections to 2030. Int J Obes (Lond).

[39] NCD Risk Factor Collaboration (NCD-RisC) (2017). Worldwide trends in body-mass index, underweight, overweight, and obesity from 1975 to 2016: a pooled analysis of 2416 population-based measurement studies in 128·9 million children, adolescents, and adults. Lancet.

[40] Ghaem H, Ghorbani M, Zare Dorniani S (2017). Evaluation of death among the patients undergoing permanent pacemaker implantation: a competing risks analysis. Iran J Public Health.

[41] Rudziński T, Ciesielczyk M, Religa W, Zebrowski M, Bednarkiewicz Z, Krzemińska-Pakuła M (2006). Elderly and patients with sick sinus syndrome have lower chances for appropriate pacemaker mode selection, according to the Polish Cardiac Society recommendations -- a single-centre retrospective analysis. Kardiol Pol.

[42] Greenspon AJ, Patel JD, Lau E (2012). Trends in permanent pacemaker implantation in the United States from 1993 to 2009: increasing complexity of patients and procedures. J Am Coll Cardiol.

[43] Mesirca P, Fedorov VV, Hund TJ, Torrente AG, Bidaud I, Mohler PJ, Mangoni ME (2021). Pharmacologic approach to sinoatrial node dysfunction. Annu Rev Pharmacol Toxicol.

[44] Lang D, Glukhov AV (2021). Cellular and molecular mechanisms of functional hierarchy of pacemaker clusters in the sinoatrial node: new insights into sick sinus syndrome. J Cardiovasc Dev Dis.

[45] Chen W, Rams D, Zając M (2024). Morphology of human sinoatrial node and its surrounding right atrial muscle in the global obesity pandemic-does fat matter?. Front Med (Lausanne).

[46] Mazurek T, Zhang L, Zalewski A (2003). Human epicardial adipose tissue is a source of inflammatory mediators. Circulation.

[47] Chen M, Wu Q (2023). Roles and mechanisms of natural drugs on sinus node dysfunction. Biomed Pharmacother.

[48] Yanni J, Tellez JO, Sutyagin PV, Boyett MR, Dobrzynski H (2010). Structural remodelling of the sinoatrial node in obese old rats. J Mol Cell Cardiol.

[49] Sonaiya S, Zevallos A, Adrales G (2024). Long-term cardiovascular disease outcomes following bariatric surgery. J Surg Res.

[50] Yeo YH, San BJ, Ahmad E, Tan MC, Sin YM, Jani M, Bloomingdale RJ (2025). Heart failure and cardiomyopathy mortality trends and disparities among obese populations: a 20-year United States study. Prev Med.

[51] Lavie CJ, Pandey A, Lau DH, Alpert MA, Sanders P (2017). Obesity and atrial fibrillation prevalence, pathogenesis, and prognosis: effects of weight loss and exercise. J Am Coll Cardiol.

[52] Boyko EJ (2013). Observational research — opportunities and limitations. J Diabetes Complications.

[53] Cai J, Li X, Wu S (2022). Assessing the causal association between human blood metabolites and the risk of epilepsy. J Transl Med.

[54] Yuan S, Larsson SC (2023). Inverse association between serum 25-hydroxyvitamin D and nonalcoholic fatty liver disease. Clin Gastroenterol Hepatol.

[55] Pierce BL, Burgess S (2013). Efficient design for Mendelian randomization studies: subsample and 2-sample instrumental variable estimators. Am J Epidemiol.

